# What drives animal responses to high severity fire? The role of functional traits

**DOI:** 10.1002/brv.70192

**Published:** 2026-06-10

**Authors:** Grace A. Vielleux, Trent D. Penman, Victoria A. Reynolds, Matthew H. Swan

**Affiliations:** ^1^ School of Agriculture, Food and Ecosystem Science, University of Melbourne Creswick VIC 3363 Australia

**Keywords:** fire severity, global systematic review, functional traits, fire response, fauna, wildfire, species' occurrence, time since fire

## Abstract

Fire regimes are changing worldwide, with increases in the frequency, extent, and severity of fires posing growing risks to biodiversity. Fire severity – the degree of habitat alteration following fire – strongly influences both immediate survival and long‐term recovery of fauna. Yet species' responses to severe fire are highly variable, shaped by traits, time since fire, and ecosystem context. We conducted a global systematic review of studies quantifying terrestrial vertebrate responses across fire‐severity gradients. Using ordinal logistic regression, we assessed the predictive value of functional traits, time since fire, taxonomy, and ecosystem type for species' abundance and occurrence after fire. Across all severity comparisons, most species showed neutral responses; however, high severity fires elicited both more negative and more positive responses than low severity fires. Species with weak shelter, canopy‐foraging habits, and preferences for closed canopies were more likely to decline after high severity fire. Negative responses diminished with increasing time since fire, highlighting opportunities for population recovery. Our findings identify functional traits that confer vulnerability to severe fire and provide a basis for predicting species' responses in unstudied taxa or regions. Targeted management, particularly within the first few years post‐fire, will be critical to safeguard susceptible species as high severity fires become more frequent.

## I. INTRODUCTION

Fire is a natural process that supports ecosystem functions and biodiversity by maintaining habitat diversity, nutrient cycling, and species interactions (He, Lamont & Pausas, 2019; Jones & Tingley, 2022). However, these responses depend on fire regimes – the patterns of fire frequency, severity, seasonality, and extent that shape ecosystems over time (Gill, 1975; Krebs *et al*., 2010). Changes in these fire regimes can profoundly affect species' survival, reproduction, and movement during and after fire events (Santos *et al*., 2022). This is a critical consideration for future conservation as fire regimes shift from historical patterns (Doherty *et al*., 2024; Kelly *et al*., 2020). Several anthropogenic drivers have changed fire regimes, including climate change (Abram *et al*., 2021), the decline of Indigenous fire stewardship (Trauernicht *et al*., 2015; Lake & Christianson, 2019), fire suppression (Brotons *et al*., 2013) and land use modifications (Marlon *et al*., 2008). As a result, fires in many parts of the world are becoming larger, more frequent, and more severe (Pausas & Fernández‐Muñoz, 2012; Halofsky, Peterson & Harvey, 2020; Wasserman & Mueller, 2023), with profound consequences for ecosystems and wildlife.

Fire severity—the degree of ecological change caused by fire, often measured as the loss or decomposition of organic matter above and below ground (Keeley, 2009)—is a key determinant of species persistence (Whelan, 1995). Fire severity shapes post‐fire ecosystem trajectories (Bassett *et al*., 2017) and these shifts in vegetation structure can alter faunal communities (Arthur, Catling & Reid, 2012). Researchers have recorded variable inter‐species responses to fire severity which are largely attributable to variation in functional traits (Violle *et al*., 2007; Batista *et al*., 2023; Ensbey *et al*., 2023). For example, habitat specialists such as the Greater Glider (*Petauroides volans* Keer, 1792), an arboreal mammal reliant on old‐growth forests with tree hollows (Pausas, Braithwaite & Austin, 1995), experience distributional shifts that can last for years after high severity fires (Campbell‐Jones *et al*., 2022). Whereas, other habitat specialists such as the North American Black‐backed Woodpecker (*Picoides arcticus* Swainson, 1832) and the Australian Flame Robin (*Petroica phoenicea* Gould, 1837) inhabit fire‐adapted ecosystems and often seek areas affected by high severity fire (Hutto *et al*., 2008; Robinson *et al*., 2014). Other species, such as the American Robin (*Turdus migratorius* Linnaeus, 1766), considered a habitat generalist (Sallabanks, 2000), appear unaffected by high severity fires (Hutto *et al*., 2016).

Assessing the impacts of fire severity requires distinguishing between first‐order (immediate) and second‐order (longer‐term) effects on fauna (Whelan, 1995; Engstrom, 2010). First‐order effects include direct mortality from exposure to smoke and heat. In contrast, second‐order population declines are mediated through changes to the habitat and resources which occur in the years following fire (Engstrom, 2010). First‐order effects of fire result in death through anoxia, burns, extreme temperatures, or smoke poisoning (Batista *et al*., 2023), with higher mortality rates recorded during high severity fires (7% mortality) compared to low severity fires (2% mortality; Jolly *et al*., 2022). This heightened first‐order mortality following severe fires is, in part, linked to species' specific responses (Keith, 2012). For animals fleeing a high severity fire, mobile species (agile mammals, fast‐flying birds, etc.) are more likely to escape the fire front (Batista *et al*., 2023; Ensbey *et al*., 2023). For animals seeking refuge, shelters like tree hollows and ground burrows can offer protection during low severity fires, but these shelters might be compromised even in severe fires. Conversely, deep underground burrows or rocky crevices may provide protection in severe fires (Banks *et al*., 2011). Despite the significance of mortality during and immediately following high severity fire, post‐fire habitat simplification and resulting risk of predation, and loss of food or shelter may have more pronounced effects on animal survival (Newsome & Catling, 1983).

Second‐order effects of fire severity on fauna are mediated through ecosystem alterations that alter cover, nest sites, and food availability (Sutherland & Dickman, 1999). These structural changes shape key mechanisms of animal recovery, including predation rates, intraspecies competition, habitat partitioning, reproductive capacity, diet, and activity (Whelan, 1995; Sutherland & Dickman, 1999). Traits such as diet, habitat, and reproductive flexibility improve species' resilience to ecological fluctuations caused by severe fire (Ensbey *et al*., 2023). However, if environmental changes are too extreme and selective pressures too intense, populations may decline or face local extinction (Fox, 1982). For instance, predation significantly influenced small mammal declines post‐fire, especially in high‐intensity burn areas (Leahy *et al*., 2016). Although many interacting factors influence animal persistence after fire, studies consistently find that fire severity, time since fire, environmental context, and species traits are key drivers of survival and recovery.

Several reviews have explored fire effects on fauna (Berlinck, Lima & Carvalho Junior, 2021), but to our knowledge, none have globally synthesised species' responses across fire severity gradients. Previous studies have focused on specific taxonomic or trophic groups (Fontaine & Kennedy, 2012; Griffiths & Brook, 2014; Geary *et al*., 2020; Mason *et al*., 2021) or explored how fire history and regime components influence community‐level metrics such as species diversity and richness (Pastro, Dickman & Letnic, 2014; González *et al*., 2022). Collectively, these reviews highlight three key themes: the critical role of time since fire in species recovery (Mason *et al*., 2021; González *et al*., 2022), the strong influence of fire severity on species responses (Fontaine & Kennedy, 2012; Pastro *et al*., 2014; Geary *et al*., 2020; Jolly *et al*., 2022), and the potential of functional traits to reveal underlying patterns (González *et al*., 2022; Mason *et al*., 2021; Santos *et al*., 2014). Despite these advances, a synthesis that integrates these elements at the species level remains lacking. We address this gap by systematically reviewing literature that quantifies the effects of fire severity on individual species' abundance or occurrence. With these data, we aim to identify species/taxa, traits, or ecosystem contexts associated with vulnerability to high severity fire, to inform conservation management strategies.

Specifically, we ask: (*i*) what are the observed trends in species' responses to fire severity? (*ii*) Does time since fire interact with severity to affect species responses? (*iii*) Do responses to fire severity vary among taxonomic classes and ecosystem types? (*iv*) Can functional traits predict species' vulnerability to high fire severity?

## II. METHODS

### (1) Systematic protocol

We followed the ecology and evolution extension of the Preferred Reporting Items for Systematic Reviews and Meta‐Analyses (PRISMA‐EvoEco) checklist (O'Dea *et al*., 2021) to ensure the reproducibility and veracity of our reporting. We created a two‐step procedure (see online Supporting Information, Fig. S1) to ensure that our systematic literature review protocol was clear and precise using a workflow crafted by (Foo *et al*., 2021) as a guide.

We employed the PICO (population, intervention, comparator, and outcome) framework (Richardson *et al*., 1995) as our guiding approach to creating an explicit selection criterion in our exploration of the research field and identification of benchmark studies (i.e. studies that are relevant and have extractable data; see Table S1 for our list of benchmark papers). We required that the research paper, dissertation, or conference paper examine terrestrial animal species exposed to fire with varying levels of severity or compare at least one severity level to an unburnt control, and report changes in each species' abundance, occurrence, density, or activity. After conducting an initial scoping search, we used the R package *litsearchr* (v1.0.0, Grames *et al*., 2018) to refine our search query (see Fig. S2 and Table S2). We evaluated the final search query to ensure it captured previously identified benchmark papers. We did not capture one benchmark paper (Hutto & Patterson, 2016) in the query because the abstract nor title specify the outcome terms; we manually included it during full‐text screening. The final query, tailored for each database – Web of Science Core Collection, Scopus, and ProQuest Dissertation and Thesis Citation Index – was composed of the following terms: (fauna* OR mammal* OR bird* OR reptile* OR amphibia* OR invertebrate* OR arthropod* OR insect* OR herpetofauna*) AND (‘fire’ OR ‘wildfire’ OR ‘burn’) AND (‘severity’ OR ‘severe’ OR ‘high‐severity’ OR ‘mixed‐severity’) AND (abund* OR ‘relative abundance’ OR ‘occurrence’ OR ‘occupancy’).

We followed methods outlined in the PRISMA eco‐evo checklist to systematically categorise and document our search process and results (Fig. 1). Before beginning our search, we registered our research plan and final search query as an embargoed project on the Open Science Framework (OSF) registry (Vielleux, 2023) to ensure research integrity and originality. We downloaded the final search query results from each database on 20 December 2023. Deduplication resulted in 717 peer‐reviewed papers, theses, and conference communications for abstract and title screening. We also incorporated all relevant literature compiled in Fontaine & Kennedy's (2012) North American meta‐analysis, as it specifically assessed the effects of fire severity on small mammals and birds. We used an online systematic review tool to streamline the reference screening process (Veritas Health Innovation, 2024). We completed the first round of screening on 31 December 2023. From the first round of screening, we included 189 studies in the full‐text review. On 19 January 2024, we limited the scope of the population criteria from all terrestrial animals to terrestrial vertebrates. This decision ensured that we could feasibly manage the volume of full‐text papers for review and extraction.

**Fig. 1:**
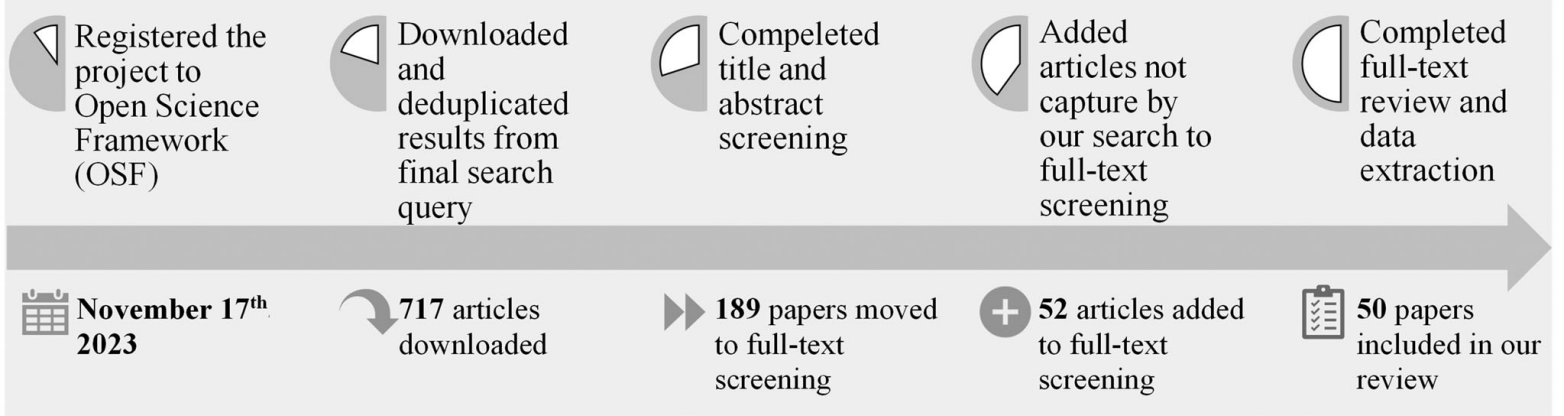
Workflow detailing the steps we undertook to process through our downloaded journal papers, reports, and theses.

During our title and abstract screening, we included only studies that assessed fire severity directly in the field or with remote sensing methods, excluding those that relied on proxy measures such as fire type or season. We also excluded studies that solely measured community metrics such as richness or diversity. We further narrowed the scope of papers during full‐text screening by selecting only those that presented extractable, analysed data in figures, tables, or text. We excluded theses with publications to prevent redundant reporting (see Fig. S3 for the diagram used to filter studies). During full‐text screening, we identified and added 11 additional papers to the title and abstract screening stage for further evaluation. We included 50 papers for our review, after screening abstracts and titles, and conducting full‐text reviews (see Appendix S1 for the citations of all included studies and Fig. S4 for the PRISMA flow diagram).

Broad extraction categories comprised of paper information (i.e. author(s) name(s), publication date, etc.), study design, site information, fire details (i.e. season of burn, fire size, etc.), method of severity classification, field techniques, species information, and individual species' population response to varying levels of severity. We recorded latitude and longitude for each study based on figures or the author's description of the location. We classified each location according to the IUCN global ecosystem typology (Keith *et al*., 2020). Although each location has attributes from several ecosystem functional groups, we only recorded the main terrestrial ecosystem functional group for each study. Additionally, we categorised each study location based on the fire regime type (fire‐dependent, fire‐independent, or fire‐sensitive) according to the global fire regime maps (Shlisky *et al*., 2009). For all papers and theses, we extracted data from figures, tables, written results, or a combination of these. We extracted all data to a Microsoft Access (Version 2407 Build 16.0.17830.20166) relational database.

We followed the guidelines outlined in the Cochrane Handbook for Systematic Reviews of Interventions (McKenzie & Brennan, 2019) to assess species' directional population changes using a vote‐counting approach. This method involved tallying the statistical significance and inferred trends reported in each study (Doherty *et al*., 2023; see Appendix S2 for a detailed description of extraction protocols). We acknowledge that conducting a vote‐counting analysis, as opposed to calculating effect sizes, involves several inherent assumptions. Notably, vote‐counting methods assign equal weight to all responses across studies, without accounting for variations in sampling design and effort (Hedges *et al*., 2009). However, due to the inconsistency and heterogeneity in reported effect measures across studies, a formal meta‐analysis was not feasible. Despite its constraints, vote‐counting provides a practical framework for synthesizing diverse findings and is a practical method for identifying broad trends across the literature (Bushman & Wang, 2009).

We recorded each species' change in occurrence, activity, or abundance across each examined severity level as an increase (+), decrease (−), or no change (0). Classifications of no change (0) indicates an absence of a statistically significant difference, as designated by the original authors. We assigned directional changes to population metrics at each level of severity measured compared to unburned control sites, or the other severity classes studied (or both). We also assigned species' response to fire severity at each time since fire year or interval specified by the researcher. Where authors displayed results in figures alone, we followed the guidelines suggested by Cumming & Finch (2005) to evaluate the statistical significance from confidence intervals or standard errors. If studies recorded both occupancy and abundance responses (three studies), we recorded occupancy results because this allowed for the inclusion of more species in one study that included both and ensured consistency across studies.

We used the following severity and time designations prior to data processing to record species' responses. Based on author's descriptions we classified severity levels as control (sites unburned during the studied fire event or sites surveyed before the fire event), low severity sites, moderate–low severity sites, moderate severity sites, moderate–high severity sites, and high severity sites. We recorded results at each specified time since the fire. Studies that sampled and reported species' response at multiple years since fire (e.g. species sampled at one and two years after fire) have separate entries recorded for each time. However, many studies recorded time as an interval (e.g. 1–5 years after the fire) when they did not report the specific time since the fire at the sampling period or when the analysis aggregated the sampling seasons. We did not contact any authors for additional data; however, we extracted data from some of the paper's supplementary figures and tables.

### (2) Data processing

From the 50 included studies, we recorded 1639 responses in our database. However, we consolidated time and severity categories to create comparable records resulting in 1436 responses from 327 unique species.

Our raw data included 14 potential severity categories due to inconsistent severity classifications across studies. As only a few studies assessed intermediate fire severity comparisons (e.g. 26 records comparing moderate–high *vs*. moderate severity, and 19 comparing moderate–high *vs*. low–moderate), we consolidated these categories into six broader severity groups (Fig. 2). We merged intermediate levels [low–moderate (L‐M) and moderate–high (M‐H)] into the low and high severity categories, respectively, as we determined that the extremes better represented the data. However, if entries with data from multiple comparisons had different records [e.g. a neutral response recorded at L‐M *vs*. control (C) and a positive response recorded at L *vs*. C] we consolidated the records to moderate comparisons instead (M *vs*. C or H *vs*. M). Following these consolidations, the resulting comparisons were low severity compared to unburned control (L *vs*. C; *N* = 965), moderate severity compared to unburned control (M *vs*. C; *N* = 837), high severity compared to unburned control (H *vs*. C; *N* = 1212), high severity compared to low severity (H *vs*. L; *N* = 345), moderate severity compared to low severity (M *vs*. L; *N* = 126), and high severity compared to moderate severity (H *vs*. M; *N* = 135).

**Fig. 2:**
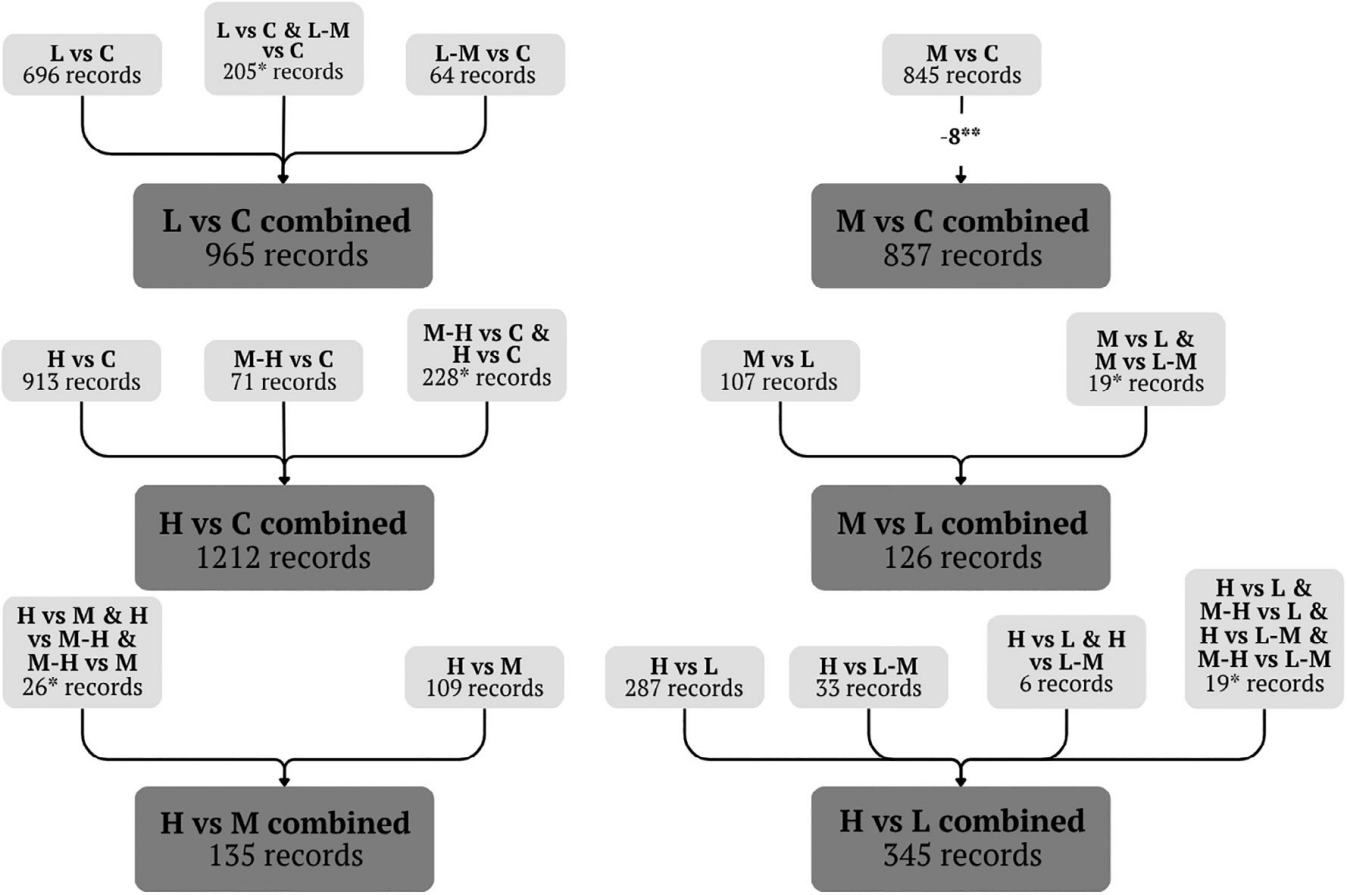
Collapsing severity comparisons from fourteen to six. * = Several shared records did not have consistent directional responses across severity comparisons (e.g. positive record at L *vs*. C and neutral record at L‐M *vs*. C), and we reclassified them to intermediary comparisons (e.g. neutral response reassigned to M *vs*. C rather than L *vs*. C). This resolved more than 80% of unmatched records (*N* = 43 unmatched; 8.5% of shared records), however eight shared records (marked with **) remained inconsistent, so we removed them from future analyses.

Data extraction resulted in 44 unique times since fire. Time was inconsistently measured across included studies with broad intervals (e.g. 2–19 years in Roberts, Burnett & Fogg, 2021), narrow ranges (e.g. 2–3 years in Chia *et al*., 2016) and discrete time points (e.g. 0.5 and 1.5 years in Kuchinke *et al*., 2020) which precluded the meaningful treatment of time as a continuous variable. To make these heterogeneous time frames comparable while retaining fidelity to the published data, we averaged all times reported as intervals to a single unit (i.e. 1–2 years since fire was averaged as 1.5). We then consolidated time into short (0–3; *N* = 765), intermediate (4–9; *N* = 446), and long (≥10; *N* = 225) time since the fire. We chose the boundaries of time since fire categories based on important regeneration stages (Cheal, 2010; Sitters *et al*., 2014) and to ensure an even spread of data among the categories (Table 1). This reclassification resulted in some studies having multiple records within a single time interval. To address this issue, we retained the record with the earliest time point (e.g. if a study reported species responses at both 2‐ and 3‐year post‐fire, we retained the 2‐year record and excluded the 3‐year record). We chose to prioritize earlier time frames because we expect earlier records to be more directly attributable to the effects of fire.

**Table 1:** Descriptions and summary statistics for the ordinal response and categorical predictor variables (we excluded the trait predictors because the traits are described in Table 1). Counts for H *versus* C and L *versus* C represent the number of records (positive, negative, or neutral) for high *versus* control and low *versus* control, respectively. There are a total of 1212 H *versus* C records and 965 L *versus* C records. Asterisks (*) indicate that we excluded the predictor variable category from the model due to an insufficient number of observations.

Variable	Description	Categories	H *vs*. C	L *vs*. C
Taxonomy	Species' classes (we assessed the classification of each species as a mammal, amphibian, or bird)	Mammal	88	55
		Amphibian	52	0*
		Bird	1071	909
		Reptile	1*	1*
Time since fire category	We grouped each authors' recorded year(s) or interval(s) as short (0–3 years), intermediate (4–9 years), and long (≥10 years) post‐fire	Short (0–3 yrs)	586	427
		Intermediate (4–9 yrs)	403	327
		Long (≥10 yrs)	223	211
Ecosystem functional type	We assigned each record a terrestrial functional ecosystem type following the typology of Keith *et al*. (2020), based on study location. We recorded ecosystem types by overlaying study coordinates with spatial data from Zenodo (Keith *et al*., 2023) in QGIS Development Team (2024). Each functional type reflects ecosystems with shared water deficit, seasonality, temperature, nutrient availability, fire activity, and herbivory (Keith *et al*., 2020).	Tropical‐subtropical dry forest and thickets (T1.2)	80	4*
		Boreal and temperate high montane rainforests (T2.1)	914	858
		Deciduous temperate forests (T2.2)	79	72
		Oceanic cool temperate rainforests (T2.3)	3*	0*
		Temperate woodlands (T4.4)	136	31
Fire response	We classified individual species' response as positive (greater abundance or occurrence in the burned area), negative (decreased abundance or occurrence in the burned area) or neutral (no difference).	Positive (+)	226	95
		Neutral (0)	755	791
		Negative (−)	231	79

To evaluate the potential of functional traits in predicting species' responses to high severity fire, we assigned vulnerability rankings for six traits to all species assessed at high severity *versus* control conditions (*N* = 269). Table 2 shows the functional trait groups we assessed and our hypothesised vulnerability rank (0 as the lowest risk to either 2 or 3 as the highest perceived risk) to high severity fire for each trait. We sourced species trait information from open‐source trait databases (Chia *et al*., 2023; Soria *et al*., 2021; Tobias, 2022; Wilman *et al*., 2014) with additional information from websites, peer‐reviewed papers, taxonomic books, and expert knowledge.

**Table 2:** Functional traits that we used as predictors in our models. We assigned trait rankings to all species measured at high severity *versus* control sites (269 species) for a total of 1212 records across all studies and time frames. The count column shows the number of unique species associated with each trait rank.

Trait	Description	Examples	Rank	Count	Justification
Shelter strength	Strong shelter	Deep burrows, rock crevices, caves, permanent ponds, cliff banks	0	57	Fast, high‐intensity fires may trap individuals unable to escape. Primary shelter sites could inform fire resistance (Batista *et al*., 2023; Ensbey *et al*., 2023).
	Moderate shelter	Tree hollows, ephemeral ponds	1	59	
	Weak shelter	Logs, shrubs, under vegetation, exposed trees, shallow crevices	2	115	
	Exposed	Open ground, litter, shallow burrows	3	38	
Reproductive cycle	Flexible breeder	Breeds multiple times in a year	0	24	Single‐season breeders may be more vulnerable during fire seasons if pregnant or caring for young (Batista *et al*., 2023).
	Single‐season breeder	Breeds in Spring, Winter, or Autumn	1	155	
	Dry season breeder	Breeds in Summer	2	90	
Habitat preference	Open canopy	Grasslands, wetlands, clearings, rocky outcrops	0	56	Closed‐canopy species may face reduced foraging/reproductive opportunities (Moretti & Legg, 2009; Albanesi, Dardanelli & Bellis, 2014).
	Semi‐dense canopy	Woodlands, chaparral, heath	1	106	
	Closed canopy	Rainforests, dense forests	2	107	
Habitat breath	Generalist	Occupies ≥3 habitat types (IUCN, 2025)	0	126	Specialists may face slower recovery due to specific habitat requirements (Kelly *et al*., 2010; Ensbey *et al*., 2023).
	Intermediate	Occupies 2–3 habitat types	1	92	
	Specialist	Restricted to one habitat type	2	51	
Foraging strata	Generalist/Aquatic/Aerial	Varied strata or buffered strata	0	68	Severe fire may affect canopy‐foraging species more than ground‐dwelling species (Orgeas & Andersen, 2001).
	Ground/Fossorial	Ground or underground	1	103	
	Midstory	Shrubs or low vegetation	2	74	
	Canopy	Tree canopies	3	23	
Diet breadth	Generalist	Consumes ≥3 food groups	0	66	A broader diet may enhance post‐fire resilience (Ensbey *et al*., 2023; Santos *et al*., 2014).
	Intermediate	2–3 food groups	1	141	
	Specialist	Single food group	2	62	
Primary food recovery	Short	Grass, shoots, herbs, carrion, fish, buds	0	51	Recovery speed of primary food may influence population recovery (Ensbey *et al*., 2023).
	Medium/mixed	Invertebrates, small vertebrates, bark, nectar, or seeds	1	188	
	Long	Fruits, nuts, large vertebrates, or pollen	2	30	

### (3) Data synthesis and analysis

We used graphic visualisations and percentages to assess taxonomic, geographical, and methodological patterns in the literature. Where researchers provided fire information, we generated summaries of fire size, name, or season of the fire. We defined fire assessment as the broad method investigators used to detect differences in fire severity (remote or field). Additionally, we classified assessment methods by categorising the specific measurement(s) used to determine different gradients between severity classes. Remotely sensed methods are those that use satellite or aerial equipment in the assessment of fire severity, and these were further categorised by the spectral bands [i.e. normalised difference vegetation index(NDVI), differenced normalised burn ratio (dNBR)] or whether researchers used aircraft photography. Field investigations were those that required on‐the‐ground assessment to determine fire severity gradients. Because field investigation metrics used to categorise fire severity gradients were diverse across studies, we allocated metrics to five broad categories. The five aspects of the landscape used to categorise fire severity were fire patchiness, fire effects on soil and/or ground, stand mortality (metrics that assess overstorey canopy health), fire effects on stand structure (metrics that assessed multiple layers of the forest), and fire effects on individual tree characteristic (i.e. needle colour or presence of white ash). We tallied the method(s) used by papers and grouped them by continent to visualise the regional preferences in fire severity assessment and categorisation.

To answer our first research question, species responses to fire severity across all time frames and studies were synthesised. We assigned each species as a positive, negative, mixed, or neutral responder to classify species' responses to the six severity comparisons (L *vs*. C, M *vs*. C, H *vs*. C, H *vs*. L, H *vs*. M, M *vs*. L). Then we assigned a response type based on a 75% threshold. To classify a species as a positive responder to H *versus* L severity fire, the species' records must have more than 75% of records as a ‘+’. Mixed responder classification was assigned when species do not fit into positive, negative, or neutral categories because none of the proportions of ‘+’, ‘−’, or ‘o’ reach the 75% threshold (See Fig. 3 for examples from our dataset of each response classification). To explore the proportion of responses according to other thresholds, we also tested 60% and 90% thresholds (See Fig. S5). Based on these results, we determined that a 75% threshold achieved a suitable balance between accommodating interstudy discord and requiring unity to establish a species classification. We calculated the 95% confidence interval for each response across each severity comparison. We used this threshold classification for descriptive purposes for question one alone.

**Fig. 3:**
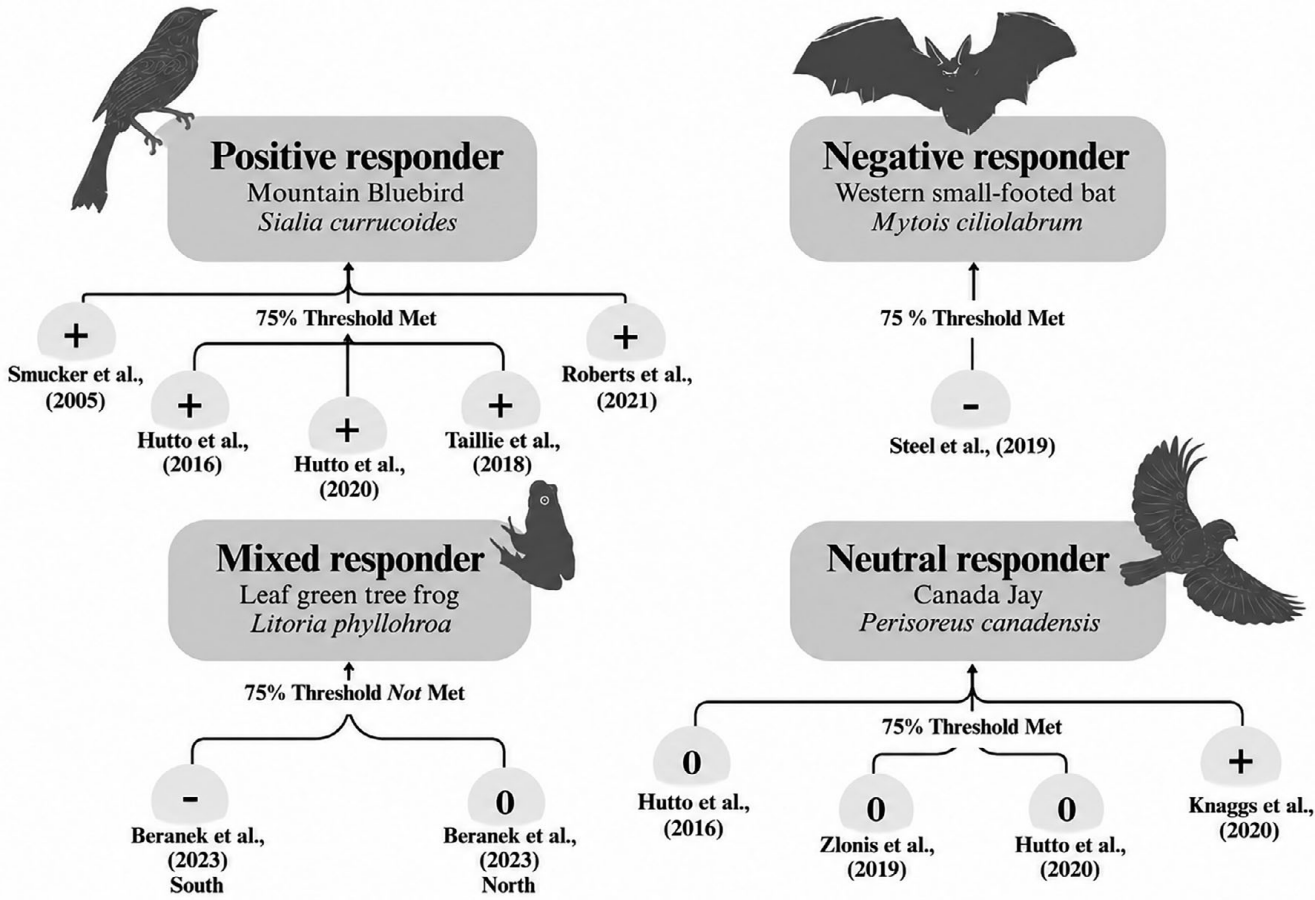
Examples of responder types from our dataset using the 75% threshold. The (+), (−), and (o) denote an increase, decrease, or neutral population change, respectively, from each source listed below the symbol. Some sources contributed more than one record (e.g. papers that measure population changes over several time steps). However, for simplicity we used the majority response from each source to visualise our methods. Beranek *et al*. (2023) is a unique case in which we counted the North and South locations as two separate responses.

To assess the effects of taxonomy, time since fire, habitat type and each trait on species responses, we used an ordinal mixed effect model, the cumulative link mixed model (clmm). The response variable was each species' fire response of positive (+), neutral (0) or negative (−) at high severity *versus* control sites and low severity *versus* control sites. We ran models with taxonomy, time since fire and habitat type predictors separately for high severity *versus* control and low severity *versus* control. We excluded all moderate *versus* control and the between‐class comparisons (i.e. high *versus* low) due to low data availability. This resulted in 43 studies with included data for high severity *versus* control (1212 records) and 27 studies with data for low severity *versus* control (965 records). For functional trait rankings, we only tested with the high severity *versus* control comparison records. We made this decision predominantly because our primary interest is understanding which traits predict species responses to high severity fire, and because the comparison of high *versus* control had the most records of all the severity contrasts. Along with testing the functional trait predictor variables described in Table 2, we tested models including taxonomy (three levels: mammal, amphibian, bird), time since fire category (three levels: short, intermediate, long), and habitat functional type (4 levels: T2.1, T2.2, T4.4, T1.2) on species' response to fire severity (Table 1).

We conducted all analysis in R (R Core Team, 2025). Prior to modelling, we evaluated the strength of associations between predictors using Cramér's V statistic (Cramér, 1946). All correlations were below 0.34, so we retained all predictors for our analysis. We included each study's unique identifier (paper ID) as a random effect in our models to account for variability across studies and reduce the impact of study‐specific biases. We ran univariate models and compared them with null models (intercept only), assigning model fit and statistical significance using the Akaike information criterion (AIC; Akaike, 1974) and likelihood ratio tests, respectively. To identify specific group differences, we conducted pairwise comparisons with Tukey corrections at a 95% confidence level using the *emmeans* package (v1.10.5; Lenth, 2025). We generated predicted probabilities at each level of comparison from the *ggeffects* (v1.7.2, Lüdecke, 2018) package and used the *ggplot2* (v3.5.1, Wickham, 2016) package to visualise the model's predicted probability of each response (‘+’, ‘−‘, or ‘o’) for each predictor level. In cases where data limitations prevented the inclusion of certain factor levels, for example the absence of records for oceanic cool temperate woodlands in comparisons between low severity and control treatments (Table 1), we used variable‐specific null models for comparison.

## III. RESULTS

Our literature review spanned three continents—Europe, Australia, and North America—and five countries: France (2%; *N* = 1 study), Spain (4%; *N* = 2), Australia (22%; *N* = 11), Canada (4%; *N* = 2), and the U.S.A. (68%; *N* = 34). In the U.S.A, most studies were from the Western states (62% of studies), followed by the Southwest (24%), Southeast (11%), and Midwest (3%), with one‐fifth of all studies taking place in California (Fig. 4). Australian studies were conducted primarily in the temperate south‐east states of Victoria (63%) and New South Wales (36%). All papers were published between 2000 and 2023, with publication rates steadily increasing throughout the 2000s (see Fig. S6).

**Fig. 4:**
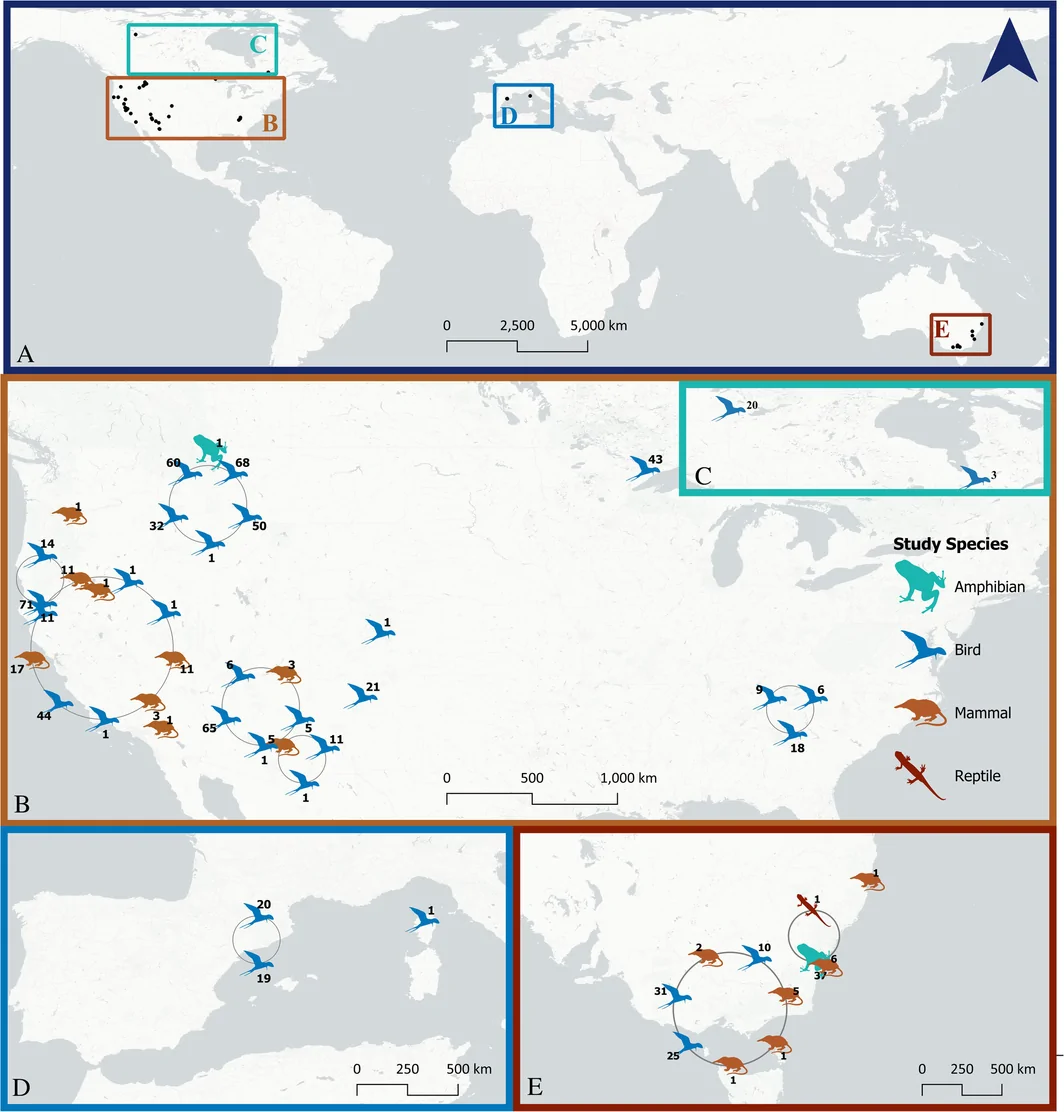
Global distribution of included studies. Map A shows the locations of all extracted study locations which are examined closer in B, C, D and E. Grey circles in B, C, D, and E denote locations where studies are clustered (larger circles indicate areas with greater density of studies). Each map is highlighted with unique colours that match the outlined areas in E. B Shows studies conducted in the United States, C in Canada, D in Spain and France, and E in Australia. The number beside each animal symbol is the count of species examined by each respective study.

Researchers assessed 101 fires across the 50 studies. The majority, 82% (*N* = 41) of studies, assessed a single fire. The median fire size was 225.6 km^2^, and the mean was 3891.8 km^2^ (39 studies reported fire size). Of the 43 studies that listed the fire type, 39 (91%) studied wildfires. Three studies examined species responses to both prescribed and wildfires, and one focused solely on prescribed burns. Six separate studies examined the Kilmore East‐Murrindindi fire complex that burned during Australia's Black Saturday in February 2009, followed by the 2014 King fire, 1996 Horseshoe‐Hochderffer fire, and the 2019–2020 Black Summer fires, each investigated by three studies.

Four taxonomic groups were represented: birds (90% of records; 72% of species; 32 studies), mammals (6% of records; 16% of species; 15 studies), amphibians (4% of records; 12% of species; 2 studies) and reptiles (1 record, 1 species, 1 study). Across all studies, 12 orders of birds (Passeriformes representing 84% of the records), nine orders of mammals (majority represented by Chiroptera and Rodentia) and chiefly the Anura order for amphibians (96% of the records) were studied (see Table S3 for the list and percentages of each order included in our review). Dreeite Water Skink (*Eulamprus tympanum* Lönnberg & Andersson, 1915) was the only reptile assessed. Among the 327 unique species studied, IUCN (2025) lists 23 (7%) species as vulnerable, near threatened, or endangered (see Table S4 for the list of all threatened and near threatened species). Over 42% (*N* = 140) of species were examined by more than one study. One‐third of the studies (*N* = 16) examined the effect of fire severity on a single species. The Yellow‐rumped Warbler (*Setophaga coronata* Linnaeus 1766) was the most frequently studied species, appearing in 13 papers. However, both the Hairy Woodpecker (*Leuconotopicus villosus* Linnaeus 1766) and the Yellow‐rumped Warbler have the highest number of records (*N* = 25) because of studies that assessed multiple time since fire years or intervals (i.e. researchers evaluated species occupancy at 1‐, 2‐ and 3‐year post‐fire).

Over half of all studies (*N* = 29) used more than one measurement to categorise severity. Field‐based methods were used in 42% of studies, remote sensing in 38%, and both methods in 20%. There was variation in how countries categorised severity: 50% of Australian studies used stand structure metrics (e.g. canopy scorch height, charring extent), compared to just 20% in the U.S.A (Fig. 5). The most used metric in U.S studies was the differenced normalised burn ratio (dNBR; 29.1%), followed by stand mortality (27.3%). In Canada, all studies used remote methods, equally split between aerial imagery (to estimate canopy mortality) and satellite‐based dNBR. Field methods in France and Spain differed from the other countries, using unique indicators such as the smallest pine crown scorch length (France) and fire patchiness (Spain).

**Fig. 5:**
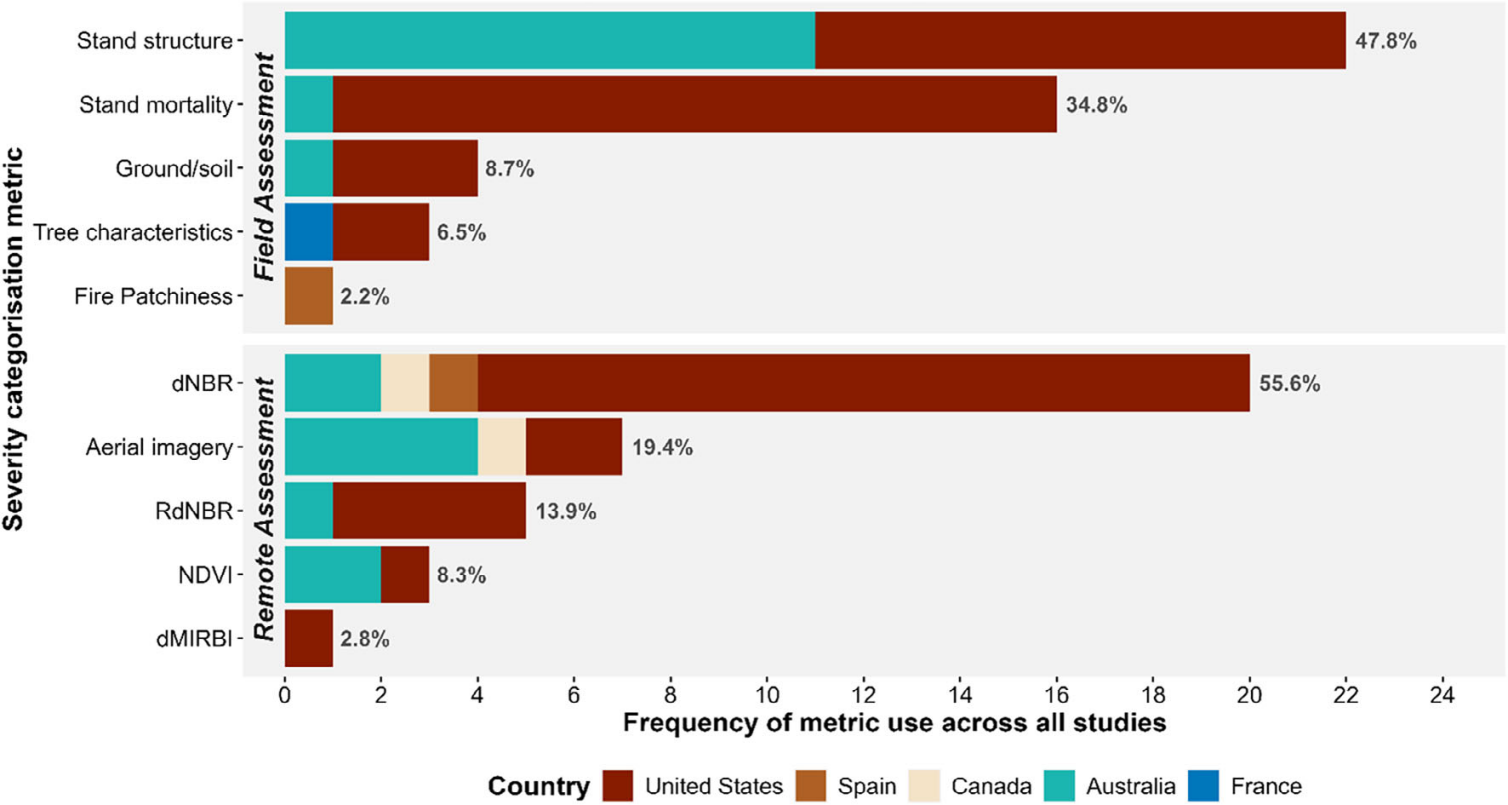
Fire severity assessment and categorisation by country and assessment method. Severity metrics are grouped by assessment method (field‐based and remotely sensed). Bars show the frequency of use of each metric across all included studies, with colours indicating contributing countries (United States, Spain, Canada, Australia, and France). Percentage labels denote the proportion of total metric use within each assessment method. RdNBR = relative differenced normalised burn ratio; NDVI = normalised difference vegetation index; dNBR = differenced normalised burn ratio; dMIRBI = differenced mid‐infrared bi‐spectral index.

### (1) What are the observed trends in species' responses to fire severity?

The majority of species showed a neutral response to all the assessed severity comparisons across all studies and time frames (Fig. 6). High severity resulted in more positive, negative, and mixed responses from species than low severity. A higher proportion of species are classified as negative responders in high severity *versus* control sites (10%, *N* = 27 species) and in high severity *versus* low severity sites (15%, *N* = 17) compared to only 5% of species (*N* = 8) in low severity *versus* control sites. Similarly, high severity compared to unburned and low severity both have a greater proportion of positive responders, 7%, compared to 2% of species with responses measured between low severity *versus* control. The comparisons between high and moderate severity and moderate and low severity both demonstrate the highest proportion of neutral responders, 83% and 94%, respectively. Mixed responders, species whose culmination of records across studies has no unified response, are highest (30%, *N* = 78) in high severity *versus* control sites.

**Fig. 6:**
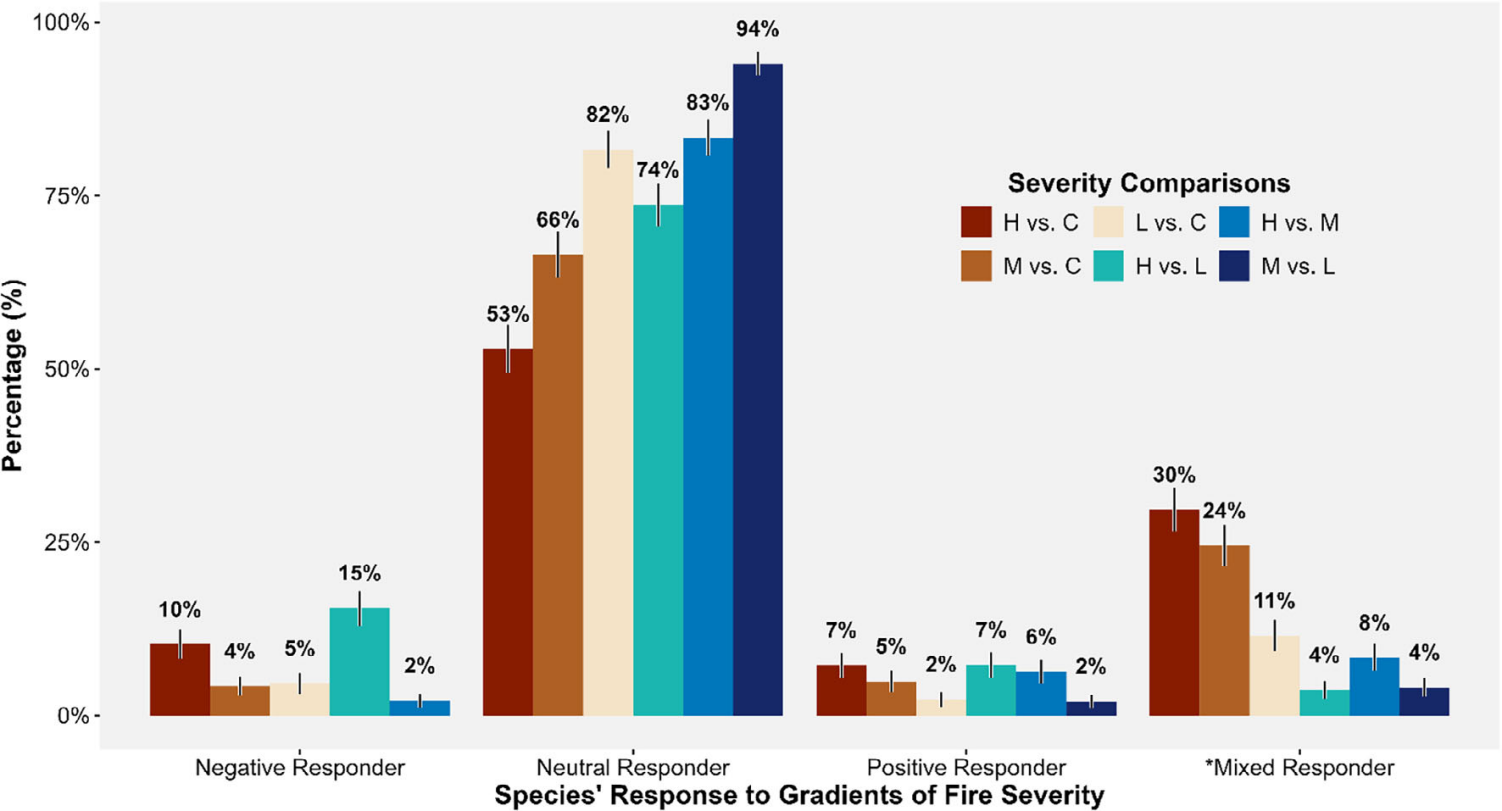
Proportional display of species' responses to gradients of fire severity across all times and studies. We classified all responses based on a 75% threshold of each individual species' total recorded responses. Error bars represent 95% confidence intervals. H = High severity, L = Low severity, M = Moderate severity, C = Unburned comparison. * = Mixed responders represent species that have more than one record with no proportion of species' records ‘+’, ‘−’, or ‘o’ reaching the 75% threshold.

### (2) Does time since fire interact with severity to affect species' response to fire severity?

For high severity *versus* control comparisons, the model that included time since fire was statistically different from the null model (*χ* ^2^ (2) = 17.97, *P* < 0.001). The model fit was improved when it included time since fire compared to the null (ΔAIC = 14). Post‐hoc pairwise comparisons highlighted that short (0–3 years) time since fire had lower species response values compared to both intermediate (4–9 years; estimate = −0.551, *P* < 0.01) and long (≥10 years; estimate = −0.701, *P* < 0.01) time since fire. We detected no statistically significant difference between intermediate and long time since fire (estimate = −0.15, *P* = 0.75). In contrast to high severity, there was no significant difference between the low *versus* control models that included time since fire and the null model (*χ* ^2^ (2) = 3.6873, *P* = 0.16). Including time since fire did not improve model fit compared to the null model for low severity *versus* control data (ΔAIC = −0.3).

Visualizing the results of both model predictions revealed that in the first 3 years after fire, species had a higher proportion of negative responses following high severity, 22.8% [95% CI (17.5%, 28.2%)], than low severity, 7.0% [95% CI (3.2%, 10.8%)]. However 10 years post fire the predicted probability of a negative response to high severity drops to 12.9% (95 CI [7.9%, 17.8%]) (Fig. 7). This decrease in predicted probabilities of negative responses to high fire severity corresponds with a rise in the predicted probability of positive responses to high severity fire [22.5%, 95% CI (14.9%, 30.1%)] 10 years after the fire (Fig. 7). There were no differences among the three timeframes for the contrast between low severity and control.

**Fig. 7:**
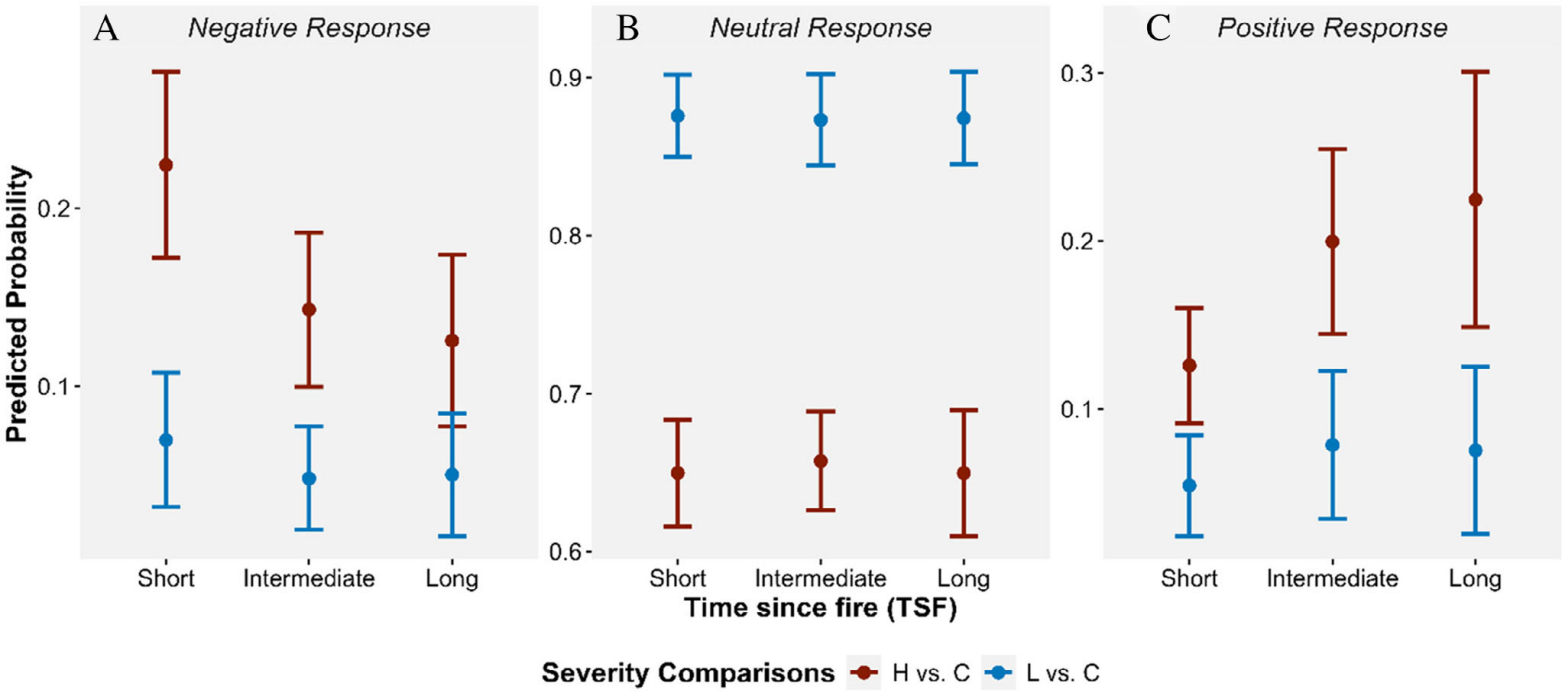
Predicted probability of species' responses to fire severity gradients with time since fire (short = 0–3 years; intermediate = 4–9 years; long = ≥10 years) from low (L) *vs*. control (C) and high (H) *vs*. C ordinal models. Note: each response, (A) negative, (B) neutral, and (C) positive, is visualised separately with a variable y‐axis to make results clearer. Error bars represent 95% confidence intervals.

### (3) Do responses to fire severity vary among taxonomic classes and ecosystem types?

The inclusion of taxonomic class as a predictor resulted in no statistically significant differences for either the high *versus* control (H *vs*. C) comparison (*χ* ^2^ (2) = 1.35, *P* = 0.51) or low *versus* control (L *vs*. C) comparison (*χ* ^2^ (1) = 1.49, *P* = 0.22) compared to each respective null model. The AIC for the taxonomic class model was slightly higher than that of the null model in both cases (H *vs*. C: ΔAIC = 2; L *vs*. C: ΔAIC = 0.5), suggesting that taxonomic class does not account for meaningful variation in species responses.

The addition of ecosystem functional types resulted in a marginal improvement over the null model for the H *versus* C comparison (*χ* ^2^ (3) = 6.67, *P* = 0.08), but the AIC difference (ΔAIC = 0.7) was insufficient to support a meaningful increase in model fit. We found no significant difference between the null and ecosystem type models for the L *versus* C comparison (*χ* ^2^ (2) = 2.92, *P* = 0.23), with the predictor model showing a higher AIC compared to the null (ΔAIC = 1.1). Predicted probabilities from each time since fire, taxonomy and habitat functional type model are in the supporting information (Figs S7–S10).

### (4) Can functional traits predict species' vulnerability to high fire severity?

Five of the seven traits modelled had statistically detectable differences from the null model. Species' habitat preference was the strongest predictor of species' response to high fire severity (*χ* ^2^ (2) = 133.09, *P* < 0.0001) and supported the best model fit according to AIC reductions (ΔAIC = 129.1). Pairwise comparisons were statistically significant between all contrasts (*P* < 0.001). Species associated with closed canopy habitats had a higher predicted probability of a negative response by 31% (95% CI: 22.8%–39.2%) compared to 5% (95% CI: 3%–12%) of species that prefer open canopies (Fig. 8C).

**Fig. 8:**
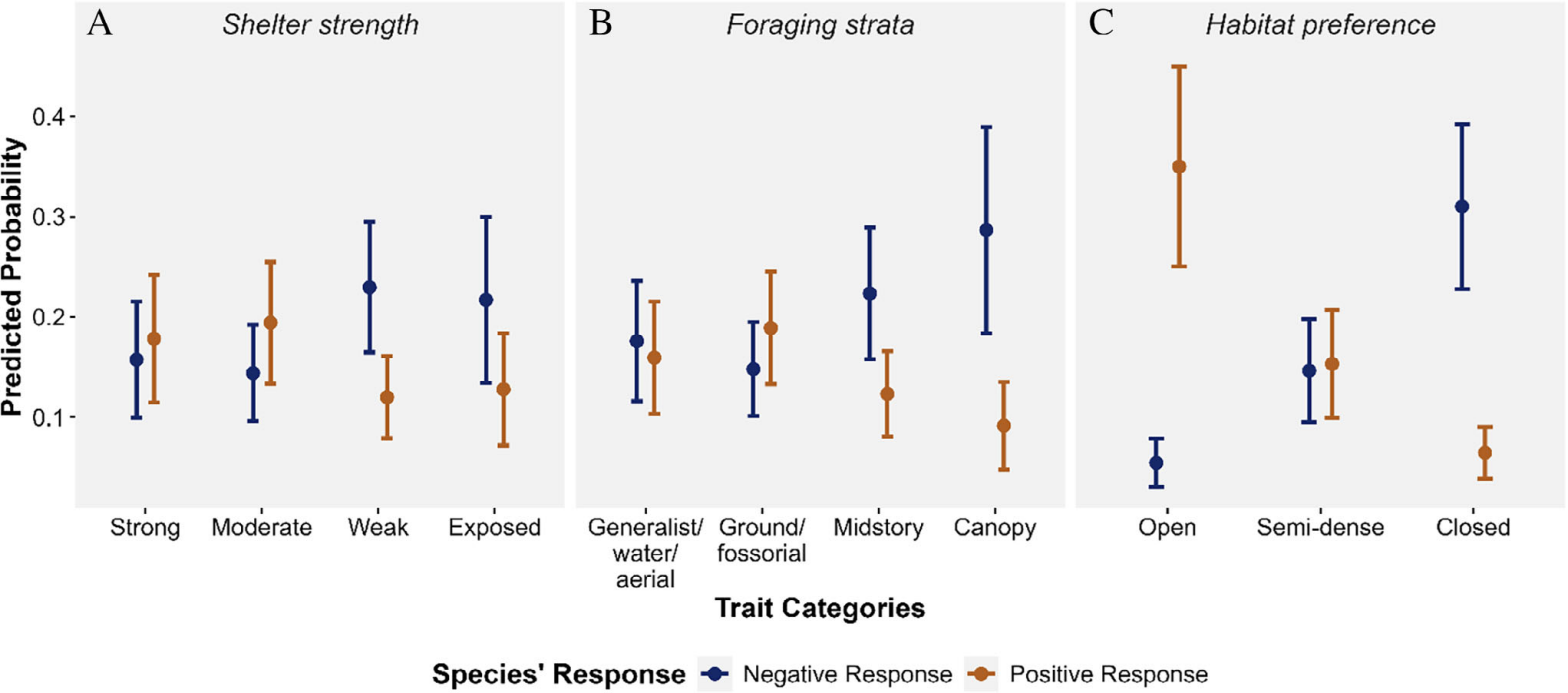
Ordinal model outputs of predicted probabilities of positive and negative species' responses across each trait category for (A) shelter strength, (B) foraging strata and (C) habitat preference. Error bars are 95% confidence intervals. Note: We have not shown neutral responses to make the effects of traits on negative and positive responses more visible.

Foraging strata and shelter strength were both statistically significant predictors of the response variable (foraging strata: *χ* ^2^ (3) = 20.62, *P* < 0.0001; shelter strength: *χ* ^2^ (3) = 18.32, *P* < 0.0001) and supported improved model fit (ΔAIC = 14.6 and 12.3, respectively). For shelter strength, species that use moderate shelters were less likely to respond negatively to high severity fire than those using weak shelters (*P* < 0.05), with predicted negative response probabilities of 14.4% (95% CI: 9.6%–19.2%) *versus* 22.9% (95% CI: 16.5%–29.4%), respectively (Fig. 8A). Pairwise comparisons for foraging strata were different (*P* < 0.05) for ground *versus* both midstory and canopy dwellers. Ground dwellers had the lowest predicted probability of a negative response (14.8%; 95% CI: 10.1%–19.5%), followed by generalist/ aerial/aquatic species (17.6%; 95% CI: 11.6%–23.6%), midstory dwellers (22.3%; 95% CI: 15.8%–28.9%), and canopy dwellers (28.7%, 95% CI: 18.4%–39%) (Fig. 8B).

Both reproductive cycle and habitat breadth were statistically significant predictors of the response variable, with models including each variable showing detectable differences from the null model (reproductive cycle: *χ* ^2^ (2) = 9.76, *P* < 0.01; habitat breadth: *χ* ^2^ (2) = 8.66, *P* < 0.01). These models also demonstrated improved model fit based on reductions in AIC (ΔAIC = 5.8 and 4.7, respectively). However, *post hoc* pairwise comparisons of estimated marginal means revealed that only certain levels within these traits were statistically distinct. Specifically, dry‐season breeders and habitat specialist were both 1.4 times more likely to have a negative response than single‐season breeders and habitat generalists, respectively (see Figs S11 and S12 for predicted probabilities of habitat breadth and reproductive cycle).

Food recovery and diet breadth were not statistically significant predictors of the response variable. Neither model showed statistically detectable differences from the null (food recovery: *χ* ^2^ (2) = 1.49, *P* = 0.47; habitat breadth: *χ* ^2^ (2) = 4.42, *P* = 0.11; see Figs S13 and S14 for model predicted probabilities).

## IV. DISCUSSION

### (1) Species responses to the effects of fire severity

High severity fire is becoming more frequent worldwide (Wasserman & Mueller, 2023), yet its impact on terrestrial vertebrates is far from uniform. Our global synthesis of 327 species provides a novel quantitative, cross‐continental assessment of responses to fire severity. Overall, we found a high degree of ecological resilience: more than half of species were classified as neutral responders, consistent with other recent studies (Calhoun *et al*., 2023; Pocknee *et al*., 2023; Linley *et al*., 2024). However, responses at high severity sites showed greater polarisation, with elevated proportions of both positive and negative outcomes. This pattern echoes disturbance ecology theory, which predicts that intense disturbances amplify species' ecological strategies, creating clear ‘winners’ and ‘losers’ (Miller, Roxburgh & Shea, 2011; Andersen, 2021).

To identify species with robust, repeatable responses, we examined those assessed in at least two independent studies (*N* = 140). Five species consistently responded positively to high severity fire: the Western Bluebird (*Sialia mexicana* Swainson, 1832), Mountain Bluebird (*Sialia currucoides* Bechstein, 1798), Hairy Woodpecker (*Leuconotopicus villosus* Linnaeus, 1766), Lazuli Bunting (*Passerina amoena* Say, 1823), and Eastern Towhee (*Pipilo erythrophthalmus* Linnaeus, 1758). These species generally prefer open‐canopy habitats or are habitat generalists (Bent & Austin, 1968; Morimoto & Wasserman, 1991; Johnson & Wauer, 1996; Mills, Rumble & Flake, 2000). Whereas all negative responders – Red‐breasted Nuthatch (*Sitta canadensis* Linnaeus, 1766), Gray Flycatcher (*Empidonax wrightii* Baird, 1858), Hutton's Vireo (*Vireo huttoni* Cassin, 1851), Rose Robin (*Petroica rosea* Gould, 1840), and Cordilleran Flycatcher (*Empidonax occidentalis* Nelson, 1897) – prefer closed canopy, are exposed during fires, forage exclusively in the canopy, or exhibit a combination of these tendencies (Russell & Woodbury, 1941; Fitzpatrick, 1981; Recher *et al*., 1985; Hutto, 1985; Adams & Morrison, 1993). Birds dominated both positive and negative responder lists, reflecting the strong research bias toward avian taxa in fire severity studies. When including all positive responders, 14 of the 16 species were birds, with only two mammals – the Silver‐haired Bat (*Lasionycteris noctivagans* La Conte, 1831) and Western Gray Squirrel (*Sciurus griseus* Ord, 1818) – also showing positive responses. Negative responders show a more diverse assemblage. Of the 29 species classified as negative responders, seven are mammals (24% of the 29 species), five are amphibians (17%), and one is the only reptile, the Dreeite Water Skink, captured in our review (3%). These proportions of mammals, and amphibians are notably higher than their respective representations of 16%, and 12% across all included species (*N* = 327; note that since there was only one reptile species, it would overrepresent whichever category it fell into). This suggests that taxonomy, though not a significant predictor in our analysis, may still be a valuable indicator of species' vulnerability to fire. Nevertheless, we highlight that these consistent responses clearly illustrate that certain species exhibit strong, directional responses to high severity fire. We suggest these patterns reflect the varying sensitivities and adaptations among species, as illustrated by their compendium of functional traits.

### (2) Contextualising ‘mixed responders’

A substantial proportion of species – 30% and 24% for high‐ and moderate‐severity, respectively – were classified as mixed responders. We propose that methodological differences, ecological disparities, and divergent fire histories are the primary factors contributing to mixed response classifications. We also acknowledge that in our classification system only species studied in more than one paper can be mixed responders, so better studied species are more likely to be classified as mixed responders.

Variances in study design and outcome measures are prominent sources of dissonance across all studies (see Tables S5 and S6 for proportions of study design and outcome measures, respectively). Such differences can significantly affect the detection and interpretation of ecological changes (Christie *et al*., 2019), potentially distorting observed biodiversity responses (Pollock *et al*., 2002; Christie *et al*., 2020). Besides variations in study design, different methods of severity assessment may contribute to mixed responses. Differences in the accuracy and relevance of severity metrics may influence how studies document individual species' responses. While remote sensing offers advantages such as rapid assessment, access to difficult terrain, and accurate detection of canopy change (Cohen & Spies, 1992) relying solely on remote sensing can be unreliable for detecting differences across various forest heights (Hammill & Bradstock, 2006) and among vegetation types (Gale & Cary, 2022). To accurately capture how fauna respond to gradients of fire severity, incorporating field measures that assess key habitat structures is important for validating remotely sensed fire severity (Cansler & McKenzie, 2012; Densmore *et al*., 2023).

Mixed responses may be related to variation in how species respond to fire across climatic gradients or in different ecosystems. For example, Beranek *et al*. (2023) studied 14 frog species following the 2019–2020 Black Saturday fires in Australia, finding that four species responded to fire severity differently dependent on whether they were North or South of the Great Dividing Range. Gradients of fire severity create different thermal microclimates for frogs (Hossack *et al*., 2009); and the influence of fire severity gradients on forest microclimates differs between forest types, with wetter systems exhibiting greater fluctuations than drier systems (Wolf *et al*., 2021). This aligns with findings of fire severity eliciting greater variability in species' responses in the northern–wetter sites than southern–drier sites (Beranek *et al*., 2023). Future research that assesses finer‐scale ecosystem differences may find more conclusive evidence of how different vegetation types interact with fire severity to impact biodiversity outcomes.

Furthermore, other elements of fire regimes such as fire size may have influenced the consistency of species responses to fire severity. For example, the Bush rat (*Rattus fuscipe*s Waterhouse, 1839) was assessed by two independent studies that reported divergent responses – positive (Chia *et al*., 2016) and negative (Lindenmayer *et al*., 2016) – to high severity fire in the first 2 years following fire. The key difference between studies was fire size and history. Chia *et al*. (2016) studied responses to a fire 80 times larger than the one examined by Lindenmayer *et al*. (2016) and sites examined by Lindenmayer *et al*. (2016) generally had a history of frequent fire and a median time since last fire that was lower than sites assessed by Chia *et al*. (2016). Investigating interactions among fire severity and other fire regime elements is a key area of future research.

In addition to mixed responders, a substantial proportion of species were classified as neutral responders, indicating a high level of resilience to fires of different severities. This outcome could be interpreted as an absence of species responses to high severity fire. However, neutral classifications reflect cases where observed effects were statistically non‐significant (i.e. no directional change), as designated by the original studies. Such outcomes may arise because true responses are small in magnitude, or because the available data lacked sufficient statistical power to detect effects. Consequently, species classified as neutral may still respond positively or negatively to fire, but in ways that are subtle or context‐dependent. This highlights the importance of interpreting neutral responses cautiously, particularly in a synthesis spanning diverse taxa, ecosystems, and study designs.

### (3) Effects of time since fire

Many studies have used time since fire to predict faunal recovery (Fox, 1982; Nimmo *et al*., 2012; Watson *et al*., 2012), based on the assumption that vegetation – thus habitat – recovers predictably over time (Clarke, 2008; Kelly, Brotons & McCarthy, 2017). Yet time since fire alone is not a sufficient surrogate to predict species' responses accurately (Sitters *et al*., 2014). Other researchers highlight habitat structural development over time as a superior alternative (Monamy & Fox, 2010; Swan *et al*., 2015). Fire severity in combination with time since fire changes the trajectory of habitat structure recovery (Freeman & Kobziar, 2011; Etchells *et al*., 2020) and improves model accuracy (Taillie *et al*., 2018; Swan *et al*., 2018). Our results reinforce findings that fire severity and time since fire interact in meaningful ways. Interestingly, we observed no difference between intermediate (4–10 years) and long (≥10 years) time since fire for high severity burn sites. This may indicate a general plateauing of ecological recovery after 4 years since fire. Conversely, this lack of a notable difference between intermediate and long time since fire may reflect the fact that post‐fire succession proceeds at different rates and along different trajectories across diverse climates and vegetation communities (Luo *et al*., 2022). Such context‐dependent variation may obscure true differences between intermediate and long recovery periods.

High severity sites experienced more negative responses from animals compared to unburned sites in the short term (0–3 years post‐fire). This immediate decline could be due to direct mortality from fire (Woolley *et al*., 2008). However, high fatality rates directly caused by the fire event are not ubiquitous. Low rates of direct fire mortality have been reported by several researchers (Bond *et al*., 2002; Fenner & Bull, 2007; Garvey *et al*., 2010; Nowack, Cooper & Geiser, 2016), even from severe fires (Banks *et al*., 2011), however there is a notable lack of studies that have assessed direct mortality following severe wildfires (Jolly *et al*., 2022). We suggest that aside from direct mortality, migration to unburnt fire refugia (Garvey *et al*., 2010; Robinson *et al*., 2014; Shaw *et al*., 2021) or increased movement in areas burnt at lower severity (Volkmann & Hodges, 2021; Lane *et al*., 2024) may contribute to our observed increase in short‐term negative responses. Additional factors, such as a decrease in reproductive output following a fire (Murphy *et al*., 2010) or an increase in predation rates (Leahy *et al*., 2016), could also contribute to population changes after high severity fires. Taken together, multiple demographic processes (birth, death, and migration) may collectively explain the increased negative responses we observed in the short‐term following a fire.

As time progresses, we observe a notable transition: the prevalence of negative responses diminishes, concurrently with an increase in positive responses. Longer time since fire (greater than 10 years) elicited double the number of positive responders (*N* = 14 species) than negative responders (*N* = 6). Studies in North America found that many bird species show delayed positive responses to high severity fire (Fontaine & Kennedy, 2012; Hutto *et al*., 2016). This suggests that the benefits of high severity may require time to fully manifest. We suggest this could be because important resources take time to develop after severe fire (Berry *et al*., 2016). Additionally, population recovery may be delayed by slow *in situ* recovery (Hale *et al*., 2022) or availability of unburnt areas for recolonisation (do Rosário & Mathias, 2007; Puig‐Gironès, Clavero & Pons, 2018).

Overall, our results indicate a generalised trajectory of recovery at sites burned at high severity; however, we caution that our findings must be considered with the knowledge that past fire histories impart legacy effects – vegetation modification from prior fires – on the landscape (Foster *et al*., 2017). Evaluating the impact of a single severe fire will not provide sufficient context to fully understand the fire's effects on animal populations. Compounding effects of multiple fire raise important concerns for fauna, as research suggests that repeated high severity fires may push species beyond their recovery thresholds (Campbell‐Jones *et al*., 2022). In addition, species with persistent population declines, even as time since fire increases, may need special attention. For instance, within our database, Hutton's vireo consistently showed a negative response to high fire severity across all time frames. Species with lasting population decline may require closer monitoring and faster conservation management in areas burnt at high severity.

### (4) Traits that matter: predicting winners and losers

Several functional traits emerged as significant predictors of species vulnerability to high severity fire. Traits related to habitat structure and dependence – including shelter type, foraging strata, and habitat preference – showed the strongest capacity to predict species' responses. In contrast, the other assessed traits – habitat breadth, reproductive cycle, food recovery and diet breadth – were weak or insignificant predictors of responses.

Shelter strength was an important predictor of species' responses. Species using strong shelters such as caves or burrows were 1.4 times more likely to respond positively than species using less strong shelters. Strong shelters likely protect individuals from the fire front and may also provide cover from predators after the fire. This aligns with the findings of several field studies (Yarnell *et al*., 2008; Banks *et al*., 2011; Linley *et al*., 2024) which validate our findings that sheltering behaviour is a key predictor of species' survival and persistence in high severity fires.

Another key trait influencing species' responses to severe fire was foraging strata. Canopy foragers were nearly twice as likely to respond negatively to high severity fire as ground foragers. Such trends likely stem from the impact high severity fire has on canopy structure (Kane *et al*., 2013) and the subsequent delays in structural development of trees (Johnston, Dunn & Vernon, 2019) which affects groups such as arboreal mammals and birds (Scott & Korb, 2024; Green *et al*., 2024). Ecosystems with post‐fire regeneration traits that protract the recovery time of canopy structures may be especially susceptible to this (Rainsford *et al*., 2020). In addition to habitat recovery time, we suggest that arboreal life‐history strategies may also contribute to the observed patterns. Arboreal mammals, unlike other terrestrial vertebrates, tend to exhibit slower life‐history strategies: longer lifespans, lower fecundity, and delayed maturity (Beccari *et al*., 2024). These traits limit rapid recovery after major disturbances (Albaladejo‐Robles, Böhm & Newbold, 2023). Thus, both ecological dependencies and intrinsic life‐history constraints may heighten the vulnerability of canopy foragers to high severity fire.

Our most compelling finding was the predictive power of habitat preference. Species that preferentially select areas with closed canopies were 5.7 times more likely to respond negatively to high severity fire than species that prefer open‐canopy habitats. This result is consistent with Viljur *et al*. (2022), who found that taxonomic groups preferring open‐canopy conditions tend to benefit from disturbance. This is unsurprising, given that canopy loss is central to many fire severity metrics and a critical structural feature for many taxa (McElhinny *et al*., 2006; Galbraith *et al*., 2019). One potential mechanism behind this relationship is increased predation risk due to reduced canopy cover. However, predation patterns following fire are often ambiguous. A systematic review assessing the global response of predators to fire found no clear predictors of response (Geary *et al*., 2020). Field studies have both confirmed increased (Leahy *et al*., 2016; Hradsky *et al*., 2017; Geyle *et al*., 2024) and decreased (Jones *et al*., 2021; Hohnen *et al*., 2023) predation following fire. Multiple factors, including fire characteristics, predator hunting strategies, and prey mobility, influence predator success following fire (Doherty *et al*., 2022). Another plausible mechanism underlying our findings is the alteration of microclimatic conditions resulting from changes in canopy structure (Pike, Webb & Shine, 2011). Denser canopies can reduce light penetration, lower temperatures, and increase humidity at the forest floor, all of which can suppress fire activity by creating cooler, moister conditions (Keppel *et al*., 2017; Charles‐Dominique *et al*., 2018). Species that specialise in shaded, closed‐canopy habitats may not possess evolutionary adaptations to withstand high‐intensity fires, leaving them particularly vulnerable when such events occur (Ensbey *et al*., 2023). Although the specific mechanisms driving our observed patterns remain uncertain, our results underscore that habitat preference, particularly for closed‐canopy environments, is a robust predictor of species' vulnerability to severe fire.

Conversely, diet and food recovery traits were not significant predictors of species responses. This was unexpected, especially since an expert elicitation identified food availability as a key post‐fire driver of success (Ensbey *et al*., 2023). It is possible that food traits are not related to species responses because individual dietary flexibility may only emerge after disturbance; Dickman & Happold (2022) studied small mammal diet before and after a severe fire and found that even species with specialised diets could demonstrate considerable dietary flexibility. This adaptability may buffer species from immediate food‐related constraints following fire. Additionally, instances may arise where specialised food resources are not significantly affected by fire (Brantley *et al*., 2023). Alternatively, the lack of effects may stem from our use of coarse trait classifications. For instance, classifying species diet breadth using the number of food groups rather than the number of food items an individual species can eat might have precluded the significance of diet breadth as a predictor.

Our results also conveyed weak predictive capacity of habitat breadth and reproductive cycle. Although both models were a better fit than the null, contrasts between different classes (i.e. summer breeder *versus* flexible breeder) were not meaningful. These weak relationships likely reflect trait data that lack the resolution needed to capture ecological nuance. Specifically, IUCN‐based habitat breadth scores may not sufficiently distinguish species' habitat specialisations. However, we note that we decided on trait classifications that balanced the trade‐off between covering a wide range of species and the depth of detailed information available on their reproduction and habitat.

Future analyses would be enhanced by having access to expanded trait information especially continuous traits. Initially, we sought to include traits associated with fire escape or recovery capacity, such as body mass (Franklin *et al*., 2021), dispersal distance (Nimmo *et al*., 2019), hand‐wing index (for bats; Weeks *et al*., 2022), and aspect ratio (for birds; Luo *et al*., 2019). We also aimed to include traits like maximum longevity and generation length (Kosydar, Conquest & Tewksbury, 2014; Richards, Cooke & Bates, 2021). However, sparse records – especially for amphibians – limited our trait database to categorical traits. The goal of functional trait response groupings is to transcend taxonomic groupings and generalise the response of multiple species into categories that could provide an understanding of how distinct species assemblages react to disturbances (Blaum *et al*., 2011; Gallagher *et al*., 2021). Achieving this requires greater investment in documenting biological traits, including body size, diet specificity, and dispersal ability. Despite these limitations, our results prove the value of habitat‐related traits in predicting species vulnerability to high severity fires.

### (5) Current limitations and future outlooks

Our review highlights important insights into how species respond to fire severity. These insights can inform conservation strategies while also illuminating current limitations in the field and future research priorities.

Our findings point to several trait‐linked vulnerabilities that can guide targeted conservation actions. Species that forage in canopies, rely on weak shelters, or inhabit closed‐canopy forests appear especially sensitive to high severity fire. These traits can increase exposure to predators or reduce post‐fire habitat suitability. Additionally, species captured in this review may be particularly vulnerable in the early post‐fire period. Timely conservation action and assessment is therefore critical. However, we also caution that the ecological benefits of fire may take many years to manifest for some species, highlighting the importance of long‐term monitoring (Lindenmayer *et al*., 2012; Müller *et al*., 2016).

Despite the increasing volume of fire severity literature since the early 2000s (Fig. S6), pronounced spatial and taxonomic biases persist. For example, 78% of the records in our review pertain to bird species in North America. Such imbalances limit the generalisability of trait‐based predictions and likely contribute to the lack of significance in taxonomic class or ecosystem functional type. Notably, only one study assessed severity effects on reptiles, and only three examined amphibians. This is particularly worrying given that of the three endangered species captured, two were frogs, which both exhibited population declines after high severity fire. Thus, while taxonomy was not a significant predictor in our models, we caution against broad extrapolation. In addition to taxonomic bias, we acknowledge a geographic bias in the reviewed literature, which is likely influenced in part by our restriction to English‐language publications, potentially excluding relevant studies published in languages such as Chinese, Spanish, or Russian. Moreover, researchers conducted all studies in fire‐adapted ecosystems, which may limit the explanatory power of ecosystem type. Addressing these spatial and taxonomic gaps is a priority for future research, particularly in fire‐sensitive regions or areas not historically exposed to high severity fire.

One clear challenge in synthesising fire severity responses globally is the widely divergent approaches to assessing and categorising fire severity. Creating a universal severity metric is neither feasible nor desirable (French *et al*., 2008; Morgan *et al*., 2014). Rather, we suggest careful consideration of which indices are applied to different ecosystems (Cansler & McKenzie, 2012) is paramount to accurately assess the ecological impact of fire severity on fauna. In the context of assessing faunal populations following fire, we recommend broader adoption and refinement of tools such as the OzCBI (Australia Composite Burn Index; Densmore *et al*., 2023), which integrate field‐based habitat assessments with satellite‐derived severity data. In addition, we stress the importance of assessing fire severity across all strata layers, including below‐ground and understorey levels (Knox & Clarke, 2016). However, biome‐specific calibration remains essential to ensure accuracy across ecosystems (Quintero *et al*., 2025). Greater consistency in severity mapping will also improve the reliability of trait‐based vulnerability assessments.

Our findings underscore the complexity of ecological responses to fire severity, exemplified by the distinct variability in species' fire sensitivities and tolerance. Inappropriate fire regimes can take the form of both too much or too little fire, depending on system context (Kelly *et al*., 2020). Many systems and species need fire (Bond, Woodward, & Midgley, 2005), even severe fire (Hutto *et al*., 2008, 2016) while other systems are quickly losing habitat that is essential to fire‐sensitive species (Doherty *et al*., 2024; Mackey *et al*., 2021). Reconciling these contrasting needs is a pressing challenge for fauna conservation amidst changing global fire regimes.

## V. CONCLUSIONS

(1) Our global synthesis of species' responses to gradients of fire severity yielded two key findings. First, our result revealed a high proportion of species classified as neutral responders, suggesting a degree of resilience across taxa and ecosystems. Secondly, high severity fire was associated with increased positive, negative, and mixed responders. Taken together, these findings suggest that while species tolerate or recover from fire, increasing fire severity has the potential to drive substantial shifts in faunal community composition.
(2) We found an interaction between high fire severity and time since fire. Species tended to be most vulnerable in the immediate aftermath of high severity fire, particularly within the first one to three years. This suggests that conservation interventions may be most effective if implemented during the early post‐fire period. However, our results also revealed that some positive responses to severe fire emerged only after several years, highlighting the importance of long‐term monitoring to detect delayed recovery.
(3) Specific traits – including canopy foraging, use of weak shelters, and reliance on closed‐canopy habitats – were predictive of species vulnerability to high severity fire. These traits may limit survival or hinder recovery by increasing exposure to predators or reducing access to key resources. We recommend that land managers prioritise support for these species with such vulnerable trait profiles. Implementing interventions such as invasive predator control or protecting post‐fire refuges may be needed early after fire, particularly in areas burned at high severity.
(4) Our findings underscore the strong and complex influence of high fire severity on faunal species across taxonomic groups and geographic regions. However, significant knowledge gaps remain. Reptiles, amphibians, and mammals remain underrepresented in the literature on fire severity. Future research should address these taxonomic deficiencies and extend analyses to less‐studied ecosystems. We also advocate for the use of ecologically relevant fire severity metrics which will enhance the comparability of findings and support more robust global syntheses of biodiversity responses to fire.

## AUTHOR CONTRIBUTIONS

Conceptualisation: Grace A. Vielleux, Matthew Swan, Trent Penman; Data curation: Grace A. Vielleux; Formal analysis: Grace A. Vielleux, Matthew Swan, Victoria Reynolds, Trent Penman; Investigation: Grace A. Vielleux; Methodology: Grace A. Vielleux, Matthew Swan, Trent Penman; Visualization: Grace A. Vielleux; Writing – original draft: Grace A. Vielleux; Writing – review & editing: Matthew Swan, Victoria Reynolds, Trent Penman.

## Supporting information


**Fig. S1.** Systematic review workflow.
**Table S1**. Benchmark studies that we used to guide and refine the development of our search query.
**Fig. S2**. Refinement of the search terms using the *litsearchr* R package.
**Table S2**. The initial naïve search string and the refined final search string.
**Fig. S3**. The selection criteria diagram we used for both rounds of screening.
**Fig. S4**. PRISMA flow diagram of identified studies that underwent screening through to the studies included for data extraction.
**Appendix S1**. Full list of all 50 studies included in our review.
**Appendix S2**. Data extraction guide.
**Fig. S5**. Threshold classification variations.
**Fig. S6**. Figure of publications years captured in our review.
**Table S3**. Counts and percentages of the records for each order included in our review.
**Table S4**. List of near threatened, vulnerable, and endangered species included in our review.
**Fig. S7**. Predicted probabilities of species' responses to high severity *vs*. control sites categorised by taxonomy.
**Fig. S8**. Predicted probabilities of species' responses to low severity *vs*. control sites categorised by taxonomy.
**Fig. S9**. Predicted probabilities of species' responses to high severity *vs*. control sites categorised by the predominant habitat functional type.
**Fig. S10**. Predicted probabilities of species' responses to low severity *vs*. control sites categorised by the predominant habitat functional type.
**Fig. S11**. Ordinal model outputs of predicted probabilities of species' responses to high severity *vs*. control across each habitat breadth category.
**Fig. S12**. Ordinal model outputs of predicted probabilities of species' responses to high severity *vs*. control across each reproductive category.
**Fig. S13**. Ordinal model outputs of predicted probabilities of species' responses to high severity *vs*. control across each food recovery category.
**Fig. S14**. Ordinal model outputs of predicted probabilities of species' responses to high severity *vs*. control across each diet breadth category.
**Table S5**. Study design assignment and count of each.
**Table S6**. Count of each outcome measure across all studies.

## Data Availability

The dataset compiled for this study, including species‐level fire severity responses and associated trait classifications, is archived at figshare: https://figshare.com/s/bf89ef9c95be906f0126. Data were extracted from previously published sources, which are cited in the main text and detailed in Appendix S1. Code used for data processing and analysis is available in the same repository.
