# Supplementary material for: What drives animal responses to high severity fire? The role of functional traits
Source: Biol Rev Camb Philos Soc. 2026 Jun 10;101(6):2613–33. doi: 10.1002/brv.70192 (PMC13736829; doi:10.1002/brv.70192)
Supplement: Supplementary file 1 [file BRV-101-2613-s001.docx]

**Supporting information:**

**What drives animal responses to high-severity fire? The role of functional traits.**

Grace A. Vielleux, Trent D. Penman, Victoria A. Reynolds, and Matthew H. Swan

Fig. S1**.** Systematic review workflow adapted from Foo *et al*., 2021. We followed the steps to organise our workflow to ensure research clarity, focus and reproducibility. PICO = population, intervention, comparator and outcome.

Table S1. Benchmark studies that we used to guide and refine the development of our search query. We validated our final search terms to ensure inclusion of the studies listed. Notably, our final search query did not capture Hutto & Patterson (2016), despite its relevance. We excluded Ray *et al*. (2019) after narrowing the focus to vertebrates, and Leahy *et al*. (2016), while initially considered within scope, we deemed it unsuitable upon closer review.

| **Title** | **Author (year)** | **Taxonomic class** |
| --- | --- | --- |
| Small mammal responses to fire severity mediated by vegetation characteristics and species traits | Culhane *et al*. (2022) | Mammals |
| Fire severity and fire-induced landscape heterogeneity affect arboreal mammals in fire-prone forests | Chia *et al*. (2015) | Mammals |
| Prescribed burn severity has minimal effect on common bird species in a fire-prone forest ecosystem | Kuchinke *et al*. (2020) | Birds |
| Refuges for birds in fire-prone landscapes: The influence of fire severity and fire history on the distribution of forest birds | Robinson *et al*. (2014) | Birds |
| Changes in Bird Abundance After Wildfire: Importance of Fire Severity and Time Since Fire | Smucker *et al*. (2005) | Birds |
| Positive effects of fire on birds may appear only under narrow combinations of fire severity and time-since-fire | Hutto & Patterson (2016) | Birds |
| Patterns of woodboring beetle activity following fires and bark beetle outbreaks in montane forests of California, USA | Ray *et al*. (2019) | Invertebrates |
| Amplified predation after fire suppresses rodent populations in Australia’s tropical savannas | Leahy *et al*. (2016) | Mammals |

Fig. S2. We refined the search terms using *litsearchr*, an R package that objectively and reproducibly refines search queries by identifying important key words through text-mining and key word co-occurrence networks. *Litsearchr* identified 569 two-word key words used to construct a document-feature matrix. This matrix records the frequency of each term across all the input papers and is used to establish a network of interconnected search terms. The network terms are assigned strength and rank based on each term’s number of connections to other terms. Higher strength and rank indicate terms that are more prevalent across the search results. The dotted line represents the 80 percent cutoff.

Table S2**.** *Litsearchr* validated our naïve search string, however, many of the terms captured were too specific for us to include because they identified the name of the species studied, the specific survey method, or the forest type examined. Highly ranked terms typically pertained to intervention and population terms. This is critical to note because it is prognostic of the potential for missing papers that do not specify the measured outcomes in the abstract. However, from the first one hundred key words, we identified several terms, not previously considered, that we included in the final search query. We also decided to separate our intervention terms into two categories to capture papers that may not have specifically stated that they examined a “severe fire” in the abstract.

| Our **initial** naïve search string | ALL= (fauna* OR mammal* OR bird* OR reptile* OR amphibia* OR invertebrate* OR arthropod* OR insect* OR herpetofauna*)  AND  ALL= (“fire severity” OR “wildfire severity” OR “severe fire*” OR “severe wildfire*” OR “burn severity” OR “severe burn*”)  AND  ALL= (occu* OR Abundan* OR detect*) |
| --- | --- |
| Our **refined** final search string | ALL= (fauna* OR mammal* OR bird* OR reptile* OR amphibia* OR invertebrate* OR arthropod* OR insect* OR herpetofauna*)  AND  ALL= (“fire" OR "wildfire" OR "burn”)  AND  ALL= (“severity" OR "severe" OR "high-severity" OR "mixed-severity")  AND  ALL= (abund* OR "relative abundance" OR "occurrence" OR "occupancy") |

Fig. S3**.** The selection criteria diagram we used for both rounds of screening. Note the change in population criteria from all animals to just vertebrates between Round 1 and Round 2. We collectively decided to reduce the scope of the population to ensure completion of the project.

Fig. S4**.** PRISMA flow diagram of identified studies that underwent screening through to the studies included for data extraction.

Appendix S1**.** Full list of all fifty studies included in our review. Asterisk (*) indicates publications that are a thesis.

Beranek, C., Hamer, A., Mahony, S., Stauber, A., Ryan, S., Gould, J., Wallace, S., Stock, S., Kelly, O., Parkin, T., Weigner, R., Daly, G., Callen, A., Rowley, J., Klop-Toker, K., *et al*. (2023). Severe wildfires promoted by climate change negatively impact forest amphibian metacommunities. *Diversity and Distributions* **29**, 785–800.

Brunk, K.M., Gutiérrez, R.J., Peery, M.Z., Cansler, C.A., Kahl, S. & Wood, C.M. (2023). Quail on fire: changing fire regimes may benefit mountain quail in fire-adapted forests. *Fire Ecology* **19**, 19.

Buchalski, M.R., Fontaine, J.B., Iii, P.A.H., Hayes, J.P. & Frick, W.F. (2013). Bat Response to Differing Fire Severity in Mixed-Conifer Forest California, USA. *PLOS ONE* **8**, e57884. Public Library of Science.

Campbell‐Jones, M.M., Bassett, M., Bennett, A.F., Chia, E.K., Leonard, S. & Collins, L. (2022). Fire severity has lasting effects on the distribution of arboreal mammals in a resprouting forest. *Austral Ecology* **47**, 1456–1469.

Chia, E.K., Bassett, M., Leonard, S.W.J., Holland, G.J., Ritchie, E.G., Clarke, M.F. & Bennett, A.F. (2016). Effects of the fire regime on mammal occurrence after wildfire: Site effects *vs* landscape context in fire-prone forests. *Forest Ecology and Management* **363**, 130–139.

Chia, E.K., Bassett, M., Nimmo, D.G., Leonard, S.W.J., Ritchie, E.G., Clarke, M.F. & Bennett, A.F. (2015). Fire severity and fire-induced landscape heterogeneity affect arboreal mammals in fire-prone forests. *Ecosphere* **6**, 190.

Conway, C. & Kirkpatrick, C. (2010). Effect of Forest Fire Suppression on Buff‐Breasted Flycatchers. *The Journal of Wildlife Management* **71**, 445–457.

Covert-Bratland, K., Block, W. & Theimer, T. (2006). Hairy woodpecker winter ecology in ponderosa pine forests representing different ages since wildfire. *Journal of Wildlife Management* **70**, 1379–1392.

Culhane, K., Sollmann, R., White, A.M., Tarbill, G.L., Cooper, S.D. & Young, H.S. (2022). Small mammal responses to fire severity mediated by vegetation characteristics and species traits. *Ecology and Evolution* **12**, e8918.

*Donaghy Cannon, M.M. (2011). The Use of Burned Forest By Breeding Birds Following the Biscuit Fire in Southwest Oregon. Doctor of Philosophy Thesis, Oregon State University, Corvallis, Oregon, USA.

Dwyer, J. & Block, W. (2000). Effects of wildfire on densities of secondary cavity nesting birds in ponderosa pine forests of northern Arizona. In *Kieth M, Moser CF (eds) Fire and forest ecology: innovative silviculture and vegetation managements. Tall Timbers Fire Ecology Conference Proceedings* pp. 151–156. Tall Timbers Research Station, Tallahassee.

Greenberg, C.H., Moorman, C.E., Elliott, K.J., Martin, K., Hopey, M. & Caldwell, P.V. (2023). Breeding bird abundance and species diversity greatest in high-severity wildfire patches in central hardwood forests. *Forest Ecology and Management* **529**, 120715.

Horncastle, V., Chambers, C. & Dickson, B. (2019). Grazing and wildfire effects on small mammals inhabiting montane meadows. *Journal of Wildlife Management* **83**, 534–543.

Hossack, B., Lowe, W., Honeycutt, R., Parks, S. & Corn, P. (2013). Interactive effects of wildfire, forest management, and isolation on amphibian and parasite abundance. *Ecological Applications* **23**, 479–492.

Hutto, R.L. (2008). The ecological importance of severe wildfires: some like it hot. *Ecological Applications* **18**, 1827–1834.

Hutto, R.L., Hutto, R.R. & Hutto, P.L. (2020). Patterns of bird species occurrence in relation to anthropogenic and wildfire disturbance: Management implications. *Forest Ecology and Management* **461**, 117942.

Hutto, R.L. & Patterson, D.A. (2016). Positive effects of fire on birds may appear only under narrow combinations of fire severity and time-since-fire. *International Journal of Wildland Fire* **25**, 1074–1085.

Jones, G.M., Kramer, H.A., Berigan, W.J., Whitmore, S.A., Gutiérrez, R.J. & Peery, M.Z. (2021). Megafire causes persistent loss of an old-forest species. *Animal Conservation* **24**, 925–936.

Kirkpatrick, C., Conway, C. & Jones, P. (2006). Distribution and relative abundance of forest birds in relation to burn severity in southeastern Arizona. *Journal of Wildlife Management* **70**, 1005–1012.

Knaggs, M., Haché, S., Nielsen, S., Pankratz, R. & Bayne, E. (2020). Avian Response to Wildfire Severity in a Northern Boreal Region. *Forests* **11**, 1330.

Kotliar, N.B., Kennedy, P.L. & Ferree, K. (2007). Avifaunal Responses to Fire in Southwestern Montane Forests Along a Burn Severity Gradient. *Ecological Applications* **17**, 491–507.

Kotliar, N.B., Reynolds, E.W. & Deutschman, D.H. (2008). American Three-Toed Woodpecker Response to Burn Severity and Prey Availability at Multiple Spatial Scales. *Fire Ecology* **4**, 26–45.

Kuchinke, D., Di Stefano, J., Sitters, H., Loyn, R., Gell, P. & Palmer, G. (2020). Prescribed burn severity has minimal effect on common bird species in a fire-prone forest ecosystem. *Forest Ecology and Management* **475**, 118437.

*Lambert, J.S. (2015). The 2006 Warm Fire: Effects on habitat and prey species of the northern goshawk. Master’s Thesis, Colorado State University, Fort Collins, Colorado, USA.

Latif, Q., Saab, V. & Dudley, J. (2021). Prescribed fire limits wildfire severity without altering ecological importance for birds. *Fire Ecology* **17**, 37.

Latif, Q., Sanderlin, J., Saab, V., Block, W. & Dudley, J. (2016). Avian relationships with wildfire at two dry forest locations with different historical fire regimes. *Ecosphere* **7**, e01346.

Law, B., Madani, G., Lloyd, A., Gonsalves, L., Hall, L., Sujaraj, A., Brassil, T. & Turbill, C. (2023). Australia’s 2019-20 mega-fires are associated with lower occupancy of a rainforest-dependent bat. *Animal Conservation* **26**, 103–114.

Lee, D. & Bond, M. (2015). Occupancy of California Spotted Owl sites following a large fire in the Sierra Nevada, California. *Condor* **117**, 228–236.

Letnic, M., Roberts, B., Hodgson, M., Ross, A.K., Cuartas, S., Lapwong, Y., Price, O., Sentinella, N. & Webb, J.K. (2023). Fire severity influences the post-fire habitat structure and abundance of a cool climate lizard. *Austral Ecology* **48**, 1440–1453.

Lewis, J.S., LeSueur, L., Oakleaf, J. & Rubin, E.S. (2022). Mixed-severity wildfire shapes habitat use of large herbivores and carnivores. *Forest Ecology and Management* **506**, 119933.

Lindenmayer, D., Blanchard, W., Bowd, E., Scheele, B., Foster, C., Lavery, T., McBurney, L. & Blair, D. (2022). Rapid bird species recovery following high-severity wildfire but in the absence of early successional specialists. *Diversity and Distributions* **28**, 2110–2123.

Lindenmayer, D., Blanchard, W., MacGregor, C., Barton, P., Banks, S., Crane, M., Michael, D., Okada, S., Berry, L., Florance, D. & Gill, M. (2016). Temporal trends in mammal responses to fire reveals the complex effects of fire regime attributes. *Ecological Applications* **26**, 557–573.

Lindenmayer, D., Blanchard, W., McBurney, L., Blair, D., Banks, S., Driscoll, D., Smith, A. & Gill, A. (2013). Fire severity and landscape context effects on arboreal marsupials. *Biological Conservation* **167**, 137–148.

Mazzamuto, M., Mazzella, M., Merrick, M. & Koprowski, J. (2020). Fire impacts on a forest obligate: western gray squirrel response to burn severity. *Mammalian Biology* **100**, 295–303.

Mirts, H., McLaughlin, J., Weller, T., White, A., Young, H. & Sollmann, R. (2022). Bats in the megafire: assessing species’ site use in a postfire landscape in the Sierra Nevada. *Journal of Mammalogy* **103**, 111–123.

Moneglia, P., Besnard, A., Thibault, J. & Prodon, R. (2009). Habitat selection of the Corsican Nuthatch (Sitta whiteheadi) after a fire. *Journal of Ornithology* **150**, 577–583.

Nappi, A., Drapeau, P., Saint-Germain, M. & Angers, V. (2010). Effect of fire severity on long-term occupancy of burned boreal conifer forests by saproxylic insects and wood-foraging birds. *International Journal of Wildland Fire* **19**, 500–511.

Puig-Gironès, R., Brotons, L. & Pons, P. (2022). Aridity, fire severity and proximity of populations affect the temporal responses of open-habitat birds to wildfires. *Biological Conservation* **272**, 109661.

Puig-Gironès, R., Brotons, L., Pons, P. & Franch, M. (2023). Examining the temporal effects of wildfires on forest birds: Should I stay or should I go? *Forest Ecology and Management* **549**, 121439.

Roberts, L., Burnett, R. & Fogg, A. (2021). Fire and Mechanical Forest Management Treatments Support Different Portions of the Bird Community in Fire-Suppressed Forests. *Forests* **12**, 150.

Robinson, N.M., Leonard, S.W.J., Bennett, A.F. & Clarke, M.F. (2014). Refuges for birds in fire-prone landscapes: The influence of fire severity and fire history on the distribution of forest birds. *Forest Ecology and Management* **318**, 110–121.

Rose, E., Simons, T., Klein, R. & McKerrow, A. (2016). Normalized burn ratios link fire severity with patterns of avian occurrence. *Landscape Ecology* **31**, 1537–1550.

Rush, S., Klaus, N., Keyes, T., Petrick, J. & Cooper, R. (2012). Fire severity has mixed benefits to breeding bird species in the southern Appalachians. *Forest Ecology and Management* **263**, 94–100.

Smucker, K.M., Hutto, R.L. & Steele, B.M. (2005). Changes in Bird Abundance After Wildfire: Importance of Fire Severity and Time Since Fire. *Ecological Applications* **15**, 1535–1549.

Steel, Z.L., Campos, B., Frick, W.F., Burnett, R. & Safford, H.D. (2019). The effects of wildfire severity and pyrodiversity on bat occupancy and diversity in fire-suppressed forests. *Scientific Reports* **9**, 16300.

Stephens, J., Ausprey, I., Seavy, N. & Alexander, J. (2015). Fire severity affects mixed broadleaf-conifer forest bird communities: Results for 9 years following fire. *Condor* **117**, 430–446.

Taillie, P., Burnet, R., Roberts, L., Campos, B., Peterson, M. & Moorman, C. (2018). Interacting and non-linear avian responses to mixed-severity wildfire and time since fire. *Ecosphere* **9**, e02291.

Varner, J., Lambert, M., Horns, J., Laverty, S., Dizney, L., Beever, E. & Dearing, M. (2015). Too hot to trot? Evaluating the effects of wildfire on patterns of occupancy and abundance for a climate-sensitive habitat specialist. *International Journal of Wildland Fire* **24**, 921–932.

*West, J.R. (2009). The effects on small mammal abundance after a wildfire in the Warner Mountains. Master’s Thesis, San Jose State University, San Jose, California, USA.

Zlonis, E., Walton, N., Sturtevant, B., Wolter, P. & Niemi, G. (2019). Burn severity and heterogeneity mediate avian response to wildfire in a hemiboreal forest. *Forest Ecology and Management* **439**, 70–80.

Appendix S2. Data extraction guide

*Assumptions*

Firstly, we assume severity categories (the author’s assigned category or those assigned by the reviewers) are the same. For example, if one study classed moderate fire severity based on the level of tree charring, we assign the same label of moderate severity for a paper that defined moderate severity based on RdNBR (relative differenced normalised burn ratio). We placed papers that use a continuous gradient to estimate severity into categories. For each paper, we have placed the authors’ definition of severity class into one of the following categories: control, low, low–moderate, moderate, moderate–high, or high.

Secondly, statistical significance is author-defined. There are a variety of ways that authors defined statistical significance [*P*-value, Akaike information criterion (AIC) model selection, confidence intervals, etc.]. For each recorded species, the relationship was designated as significant or non-significant as marked by the primary author. However, some results are displayed in figures rather than tables or writing. For results recorded from figures, significance was determined based on the overlap of error bars or confidence intervals (see Hossack_2013 or Puig-Girones_2022 as examples).

Thirdly, we assume that population indices are interchangeable. We accepted population indices such as frequency of occurrence, relative abundance, density, and activity as broad indicators of population change metrics. We treated each of these indices, though distinct, as the synonymous indicators of population change. However, occasionally, studies would record both relative abundance and occurrence metrics. In these cases, we chose to record occurrence data.

*Table guide*

Although the paper selection process narrowed the field for papers that have extractable data, there is still extreme variability in how each study performed experiments and analysis. These differences in study design and analysis prompted the need to create a guide for data extraction from papers. For each paper, we extracted data from figures, tables, written results, or a combination of these. Sometimes, authors would mention a relationship in the text, but if the figures or tables did not support it as significant, we marked it as non-significant. Interpreting the results for each species was often study-specific, so as a general method, we always erred on the side of caution and recorded the relationship as non-significant if interpreting the results as significant was not explicit. This decision, in combination with more stringent interpretations of figures (i.e. requiring confidence interval bars not to overlap) where authors failed to explicitly state results may have inflated the proportion of neutral responses.

To ensure transparency in how we extracted data, we recorded the following information for each study: (1) Paper classification: breakdown of how papers classify severity and what feature of the paper we extracted results from (i.e. figures, tables, supplementary, or written results sections). See the table below for more details; (2) Species #: number of species with *severity* analysed; (3) Time frames: time since fire year(s) or interval(s); (4) Data extracted to: indicates which column in the database has a (+), (-), or (o) based on the study design and analysis

We recorded results in the following columns: L *vs* C (low severity site population indices compared to the control); L-M *vs* C; M *vs* C; M-H *vs* C; H *vs* C; H *vs* L; H *vs* L-M; H *vs* M; H *vs* M-H; M-H *vs* L; M-H *vs* L-M; M-H *vs* M; M *vs* L; M *vs* L-M.

*Paper Classification*

The following represent the broad classes of analysis that each paper falls into:

| **(1)** Categorical  Researchers assign each site to a specific fire severity class. Categories are assumed to be the same across papers.  Categorical papers were further classified to identify which specific contrasts were performed.  **(1a)** Control  Paper has an analysis that compares each severity category to a control, e.g. H *vs* C, L *vs* C, M *vs* C.  **(1b)** Class  Paper has an analysis that compares each severity category to each other, e.g. H *vs* L, H *vs* M.  **(1c)** Class and Control  Paper has an analysis comparing among severity categories and among severity categories and  Controls. | **(2)** Continuous  Researchers used a spectrum of increasing severity to evaluate the effect of fire severity. To enter data into our database, continuous gradients are categorised by reviewers into the extremes (H *vs* C) or (H *vs* L). Occasionally severity was measured as a continuous metric but was then separated into categorical variables. In these cases, the study was recorded as a categorical analysis. |
| --- | --- |

Additionally, papers that analysed multiple times since fire years or intervals are denoted with a +. So a continuous study that looked at 2–4 years and 5–6 years post fire is noted as 2+). Paper ID is the first author followed by the year of publication.

|  | **Paper ID** | **Paper classification** | **Species #** | **Time frame(s)** | **Data extracted** |
| --- | --- | --- | --- | --- | --- |
| 1 | Beranek_2023 | (1a) Figure | 37 | (1) | H *vs* C |
| 2 | Brunk_2023 | (1b+) Figure | 1 | (1)  (2–5)  (6-10)  (11–35) | H *vs* L |
| 3 | Buchalski_2013 | (1a) Figure | 3 | (1) | H *vs* C  M *vs* C |
| 4 | Campbell-Jones_2022 | (2) Figure, Written | 1 | (11) | L *vs* C  H *vs* C  H *vs* L |
| 5 | Chia_2015 | (1c) Figure | 1 | (2.5) | L *vs* C  H *vs* C  H *vs* L |
| 6 | Chia_2016 | (1a) Figure, Written | 5 | (2–3) | L *vs* C  H *vs* C |
| 7 | Conway_2010 | (1c) Figure | 1 | (median time since fire was 6) | L *vs* C  M *vs* C  M-H *vs* C  H *vs* C  H *vs* L  M *vs* L  M-H *vs* L  M-H *vs* H |
| 8 | Covert-Bratland_2006 | (1c+) Figure, Written | 1 | (2)  (3)  (6)  (7) | M *vs* C  H *vs* C  H *vs* M |
| 9 | Culhane_2022 | (1c) Figure, Written | 11 | (3) | L-M *vs* C  H *vs* C  H *vs* L-M |
| 10 | Donaghy Cannon_2011 | (1b+) Figure | 11 | (1)  (2)  (3) | H *vs* L-M |
| 11 | Dwyer_2000 | (1c) Table | 5 | (1) | H *vs* C  H *vs* M  M *vs* C |
| 12 | Greenberg_2023 | (1c+) Figures, Tables & Written | 18 | (1)  (2)  (3)  (4)  (5)  Or  (1–5) | L *vs* C  M *vs* C  H *vs* C  H *vs* L  H *vs* M  M *vs* L |
| 13 | Horncastle_2019 | (2) Table | 3 | (1–2) | M *vs* C |
| 14 | Hossack_2013 | (2) Table | 1 | (7–22) | H *vs* C |
| 15 | Hutto_2008 | (1c) Written | 1 | (1–19) | H *vs* C |
| 16 | Hutto_2016 | (1a+) Figure | 50 | (1–2)  (3–4)  (6–7)  (8–9)  (10–11) | L *vs* C  M *vs* C  H *vs* C |
| 17 | Hutto_2020 | (1a+) Table | 68 | (1–4)  (5–14)  (15–35) | L *vs* C  L-M *vs* C  M *vs* C  M-H *vs* C  H *vs* C |
| 18 | Jones_2021 | (1c+) Figure | 1 | (1)  (6) | H *vs* C  L *vs* C  H *vs* L |
| 19 | Kirkpatrick_2006 | (1a) Table | 11 | (2–31)  Median time since fire was 6 years | H *vs* C  L *vs* C |
| 20 | Knaggs_2020 | (1a) Table, Written | 20 | (1–2) | L *vs* C  M *vs* C  H *vs* C |
| 21 | Kotliar_2007 | (1a+) Table, Written | 21 | (1)  (2) | H *vs* C  L-M *vs* C |
| 22 | Kotliar_2008 | (1c) Figure, Table, Written | 1 | (1–2) | L *vs* C  M *vs* C  M-H *vs* C  H *vs* C  H *vs* L  H *vs* M  H *vs* M-H  M *vs* L |
| 23 | Kuchinke_2020 | (1a) Table | 10 | (0.5)  (1.5) | H *vs* C |
| 24 | Lambert_2015 | (1c) Written | 6 | (1–4) | L *vs* C  H *vs* C  H *vs* L |
| 25 | Latif_2016 | (2) Figure | 65 | (1–3) | H *vs* L |
| 26 | Latif_2021 | (2+) Figure | 60  24 (subset of species have a yearly analysis as well) | (1–3) for all 60 species  (1)  (2)  (3)  For 24 species | H *vs* L |
| 27 | Law_2023 | (1a) Written | 1 | (1) | L *vs* C  H *vs* C |
| 28 | Lee_2015 | (2) Figure | 1 | (1) | H *vs* C |
| 29 | Letnic_2023 | (1c) Written | 1 | (1–2) | H *vs* C  H *vs* L  L *vs* C |
| 30 | Lewis_2022 | (1c) Figure | 5 | (7) | L *vs* C  M *vs* C  M-H *vs* C  H *vs* C  H *vs* L  H *vs* M-H  M-H *vs* L  M-H *vs* M  M *vs* L |
| 31 | Lindenmayer_2013 | (1a) Table | 2 | (0.5–3) | H *vs* C  M *vs* C |
| 32 | Lindenmayer_2016 | (1a/c+) Supplementary  (Supplementary figure does not match written records) | 6 | (0.5) 2003  (1) 2004  (2) 2005  (3) 2006  (4) 2007  (5) 2008  (6) 2009  (7) 2010  (8) 2011  (9) 2012  (10) 2013 | M *vs* C  H *vs* C  H *vs* M |
| 33 | Lindenmayer_2022 | (1a+) Figure | 31 | (0.5–2)  (3–5)  (7–10) | H *vs* C |
| 34 | Mazzamuto_2020 | (1a) Figure, Written | 1 | (8) | L *vs* C  M-L *vs* C  M *vs* C  H *vs* C |
| 35 | Mirts_2022 | (1a) Figure | 11 | (3) | H *vs* C  L-M *vs* C |
| 36 | Moneglia_2009 | (2) Figure, Written | 1 | (1–2) | H *vs* C |
| 37 | Nappi_2010 | (1a+) Written | 3 | (6)  (8) | L *vs* C  M *vs* C  H *vs* C  H *vs* L  H *vs* M  M *vs* L |
| 38 | Puig-Girones_2022 | (1b) Figure | 19 | (1–11) | H *vs* L  H *vs* L-M  H *vs* M  H *vs* M-H  M-H *vs* L  M-H *vs* L-M  M-H *vs* M  M *vs* L  M *vs* L-M |
| 39 | Puig-Girones_2023 | (2) Table | 20 | (1–11) | H *vs* L |
| 40 | Roberts_2021 | (1a) Supplementary | 71 | (2–19) | L *vs* C  M-H *vs* C |
| 41 | Robinson_2014 | (1c) Table | 25 | (1–3) | L *vs* C  M *vs* C  H *vs* C  H *vs* L  M *vs* L |
| 42 | Rose_2016 | (1a) Table | 6 | (1–28) | H *vs* C |
| 43 | Rush_2012 | (1c+) Figure, Written | 9 | (1–2)  (3–6) | L *vs* C  M *vs* C  H *vs* C  H *vs* M  M *vs* L  H *vs* L |
| 44 | Smucker_2005 | (1a) Table | 32 | (1–3) | H *vs* C  M *vs* C  L *vs* C |
| 45 | Steel_2019 | (1a) Figure, Written | 17 | (1–13) | H *vs* C  M-H *vs* C  M *vs* C  L *vs* C |
| 46 | Stephens_2015 | (1a+) Figure | 26 | (1)  (2)  (3)  (4)  (6)  (7)  (8)  (9) | L *vs* C  M *vs* C  H *vs* C |
| 47 | Taillie_2018 | (1a) Figure | 44 | (1–15) | L *vs* C  M *vs* C  H *vs* C |
| 48 | Varner_2015 | (1c+) Figure, Written | 1 | (1) Summer  (2) Spring  (2) Fall  (3) Spring  (3) Fall | M *vs* C  H *vs* C  H *vs* M |
| 49 | West_2009 | (1b) Written | 1 | (3) | H *vs* L |
| 50 | Zlonis_2019 | (2) Table | 43 | (1–5) | H *vs* C |

*Additional methodological notes*

Some studies had duplicates of records based on additional covariates that the study examined. Before further data processing, we consolidated or removed the additions. Beranek *et al*. (2023) examined several frog species in northern and southern regions of a large Australian fire. As the authors treated the locations as independent, we also recognise the records as independent observations. Robinson *et al*. (2014) investigated long and short time intervals since the fire. We reduced the records to include only those comparing burned sites to long, unburned areas. Varner *et al*. (2015) recorded American pika (*Ochotona princeps*) occupancy multiple times a year after a fire event. This created multiple entries in the same year, so we utilised the earliest season (spring) recorded for each year. Greenberg *et al*. (2023), Latif *et al*. (2021), and Lindenmayer *et al*. (2016) all have records of time that are both interval and single time. This is because in their analysis, they included results that were distinct for each year and conducted an overview of results over the whole study time frame. To avoid duplication, we retained only single-time records and removed the intervals.

**(A)**

**(C)**

**(B)**

Fig. S5**.** Threshold classification variations. (A) results of a 60% threshold, (B) 75% threshold used in our results section, highlighted in red, (C) results of the 90% threshold. We used these figures to visualise how adjusting the threshold of response requirement would influence the proportion of response classifications.

Fig. S6**.** Figure of publications years captured in our review. There is a clear trend (R^2^ = 0.6) of an increasing number of studies published on the topic of species’ responses to gradients of fire severity.

Table S3**.** Counts and percentages of the records for each order included in our review.

| **Class** | **Order** | **Count** | **Percent** |
| --- | --- | --- | --- |
| **Amphibians** | Anura | 51 | 96.2 |
|  | Caudata | 1 | 1.9 |
|  | Squamata | 1 | 1.9 |
| **Mammals** | Chiroptera | 32 | 34.4 |
|  | Rodentia | 31 | 33.3 |
|  | Diprotodontia | 8 | 8.6 |
|  | Dasyuromorphia | 7 | 7.5 |
|  | Peramelemorphia | 6 | 6.5 |
|  | Carnivora | 3 | 3.2 |
|  | Lagomorpha | 3 | 3.2 |
|  | Artiodactyla | 2 | 2.2 |
|  | Eulipotyphla | 1 | 1.1 |
| **Birds** | Passeriformes | 1082 | 83.9 |
|  | Piciformes | 126 | 9.8 |
|  | Caprimulgiformes | 24 | 1.9 |
|  | Galliformes | 21 | 1.6 |
|  | Columbiformes | 11 | 0.9 |
|  | Cuculiformes | 7 | 0.5 |
|  | Psittaciformes | 7 | 0.5 |
|  | Falconiformes | 4 | 0.3 |
|  | Accipitriformes | 3 | 0.2 |
|  | Strigiformes | 3 | 0.2 |
|  | Bucerotiformes | 1 | 0.1 |
|  | Coraciiformes | 1 | 0.1 |

Table S4**.** List of near threatened (white), vulnerable (yellow), and endangered (red) species included in our review.

| **Class** | **Species common name** | **Binomial classification** |
| --- | --- | --- |
| bird | Spotted owl | *Strix occidentalis* |
| bird | Olive-sided flycatcher | *Contopus cooperi* |
| bird | Evening grosbeak | *Hesperiphona vespertina* |
| bird | Rufous hummingbird | *Selasphorus rufus* |
| bird | Red-legged partridge | *Alectoris rufa* |
| bird | Iberian Grey Shrike | *Lanius meridionalis* |
| bird | Woodchat Shrike | *Lanius senator* |
| bird | Gang-gang cockatoo | *Callocephalon fimbriatum* |
| mammal | Greater glider | *Petauroides volans* |
| mammal | Golden-tipped bat | *Phoniscus papuensis* |
| bird | Corsican nuthatch | *Sitta whiteheadi* |
| amphibian | Sphagnum frog | *Philoria sphagnicola* |
| amphibian | New England tree frog | *Litoria subglandulosa* |
| amphibian | Eastern owl frog | *Heleioporus australiacus* |
| amphibian | Mixophyes balbus | *Mixophyes balbus* |
| amphibian | Davies’ tree frog | *Litoria daviesae* |
| amphibian | Giant barred-frog | *Mixophyes iteratus* |
| amphibian | Pugh’s frog | *Philoria pughi* |
| amphibian | Pouched frog | *Assa darlingtoni* |
| mammal | Little brown bat | *Myotis lucifugus* |
| bird | Pilotbird | *Pycnoptilus floccosus* |
| bird | Eastern meadowlark | *Sturnella magna* |
| bird | Pinyon jay | *Gymnorhinus cyanocephalus* |

Fig. S7**.** Predicted probabilities of species’ responses (negative, neutral, or positive) to high severity (H) *vs* control sites (C) categorised by taxonomy. We excluded reptiles due to data limitations.Error bars represent 95% confidence intervals.

Fig. S8**.** Predicted probabilities of species’ responses (negative, neutral, or positive) to low severity (L) *vs* control (C) sites categorised by taxonomy. We excluded reptiles and amphibians due to data limitations. Error bars represent 95% confidence intervals.

Fig. S9**.** Predicted probabilities of species’ responses (negative, neutral, or positive) to high severity (H) *vs* control sites (C) categorised by the predominant habitat functional type (Keith *et al*., 2023) in which the study took place. Functional types with limited data were excluded. Error bars represent 95% confidence intervals.

Fig. S10**.** Predicted probabilities of species’ responses (negative, neutral, or positive) to low severity (L) *vs* control sites (C) categorised by the predominant habitat functional type (Keith *et al.,* 2023) in which the study took place. Functional types with limited data were excluded. Error bars represent 95% confidence intervals.

Fig. S11**.** Ordinal model outputs of predicted probabilities of species’ responses (positive, negative and neutral) to high severity (H) *vs* control across (C) each habitat breadth category (0 = habitat generalist, 1 = intermediate habitat requirements, 2 = habitat specialist). Error bars represent 95% confidence intervals.

Fig. S12**.** Ordinal model outputs of predicted probabilities of species’ responses (positive, negative and neutral) to high severity (H) *vs* control (C) across each reproductive category (0 = flexible breeder, 1 = single-season breeder, 2 = dry-season breeder. Error bars represent 95% confidence.

intervals.

Fig. S13**.** Ordinal model outputs of predicted probabilities of species’ responses (positive, negative and neutral) to high severity (H) *vs* control (C) across each food recovery category (0 = short recovery, 1 = medium recovery, 2 = long recovery). Error bars represent 95% confidence intervals.

Fig. S14**.** Ordinal model outputs of predicted probabilities of species’ responses (positive, negative and neutral) to high severity (H) *vs* control (C) across each diet breadth category (0 = large diet breadth, 1 = medium diet breadth, 2 = small diet breadth). Error bars represent 95% confidence intervals.

Table S5**.** Study design assignment and count of each. Not all studies explicitly stated study design methods, so we created a categorisation framework to categorise each paper. * = Not all papers explicitly stated their study design type, so we used these indicators to classify each extracted paper ** = Not all papers report results in separate time frames, so although the study design was conducted with different time since fires the results may only show a record of one-time interval

| **Study design** | **Description** | **Categorisation*** | **# of studies** |
| --- | --- | --- | --- |
| **Before-After-Control-Impact (BACI)** | Measures the response group before and after a disturbance. A control site (unaffected by the disturbance in both sampling periods) is compared to sites exposed to the disturbance. | -Sampling before the fire  -Sampling the same sites after the fire  -Some sites sampled before the fire remain unburnt after the fire to compare with sites measured before the fire that were burned | 8 (16%) |
| **Chronosequence/**  **Space-for-time** | Sites differ in age since disturbance but are otherwise similar in climate and historic land use. Sites are stratified by time since disturbance rather than repeatedly sampled over time. | -Sampling after the fire  -Sites were categorised by time since fire (TSF) and level of fire severity  -Response of species was reported in multiple TSF intervals** | 5 (10%) |
| **Longitudinal** | Measures a response group at a site multiple times over a prolonged period. | -Sampling after fire  -Sites are visited multiple times  -Response of species was monitored through several TSF intervals** | 23 (46%) |
| **Cross-sectional** | Measures a response group at a single point in time. | -Sampling after fire  -Sampling only occurred once  -Sites are categorised as affect or unaffected by the disturbance  -Response variable is recorded at one TSF interval | 14 (28%) |

Table S6**.** Count of each outcome measure across all studies

| **Outcome measure** | **# of studies** |
| --- | --- |
| **Abundance** | 14 (28%) |
| **Activity index** | 1 (2%) |
| **Occurrence** | 31 (62%) |
| **Density** | 4 (8%) |
